# Seasonal variability of coccolith fluxes in sediment traps of the Perdido and Coatzacoalcos regions in the Southern Gulf of Mexico

**DOI:** 10.1371/journal.pone.0326673

**Published:** 2025-11-05

**Authors:** Felipe de Jesús García-Romero, Juan Carlos Herguera García, Jörg Bollmann, José Rubén Lara Lara, Mara Yadira Cortés Martínez, Amaru Márquez-Artavia

**Affiliations:** 1 Division of Oceanography, Center for Scientific Research and Higher Education of Ensenada (CICESE), Ensenada, Mexico; 2 Department of Earth Sciences, University of Toronto, Toronto, Ontario, Canada; 3 Department of Earth Science, Autonomous University of Baja California Sur, La Paz, Mexico; 4 Escuela de Ciencias Biológicas, Universidad Nacional de Costa Rica, Heredia, Costa Rica; University of Palermo: Universita degli Studi di Palermo, ITALY

## Abstract

We present new results of the coccolith fluxes in the Perdido and Coatzacoalcos areas of the Gulf of Mexico (GoM) and explore the environmental variables that may control them. The deep-water region of the GoM is known for its oligotrophic, nutrient-limited surface waters, which are relatively isolated from eutrophic waters near the coast; however, it is seasonally affected by nutrient-rich plumes of coastal waters that increase export production. Two sediment trap moorings located at a water depth of 1100 m collected settling particles from June 2016 to July 2017. The Perdido trap collected 47 species of coccoliths, and the Coatzacoalcos trap 56 species throughout the study period. Total coccolith fluxes showed a seasonal response in both trap locations, with lower fluxes during spring and summer, associated with highly stratified water column conditions that were evident in the Coatzacoalcos trap, and higher fluxes during late autumn and winter, associated with deepening mixed layer in response to cooling and to the strong “Nortes” winds. The Perdido trap showed higher total coccolith fluxes with an annual average of 3.1 x 10^9^ ± 0.9 x 10^9^ coccoliths per m^-2^d^-1^, than the Coatzacoalcos trap of 1.9 x 10^9^ ± 1.1 x 10^9^ coccoliths m^-2^d^-1^. The upper photic zone (mainly, *Emiliania huxleyi* and *Gephyrocapsa oceanica*) showed high fluxes throughout the study period in both traps, reflecting the coastal shelf influence. Overall, three species dominated the composition of the coccolith fluxes in both areas: *E. huxleyi*, *G. oceanica*, and *Florisphaera profunda*, reaching 88% in the Perdido and 84% in the Coatzacoalcos trap. These results suggest that the coccolith export production in the Perdido and Coatzacoalcos traps is strongly influenced by the cooling and deepening of the mixed layer depth during autumn and winter, as well as advection processes between the continental shelf and the offshore region, and multifactorial processes such as loop current mesoscale eddies that affect the GoM.

## Introduction

Coccolithophores are marine primary producers covered by calcareous plates called coccoliths that live in the ocean’s photic zone and play a crucial role in the marine carbon cycle [1]. They show higher abundances in nutrient-enriched conditions associated with the physical dynamics in high-latitude regions [2], where r-selected opportunistic taxa dominate the community [3]. In contrast to higher diversity patterns, more strongly associated with stratified oligotrophic settings, with k-selected species better adapted to compete effectively for scarce nutrients in stable environments conditions [4], mostly found in tropical regions and subtropical gyres [2,3]. At the end of their life cycle or when grazed by zooplankton, coccoliths are transported to the ocean floor in fecal pellets or marine snow that can be captured and analyzed in sediment traps that provide insights to the seasonal to inter-annual variability of the coccolithophores export flux to the deep ocean [5–9]. One of the main assumptions of these sediment trap studies is that the settling particles are primarily influenced by production occurring in the overlying photic zone [10–12]. However, information about the variability of coccolith fluxes is still limited in some regions, such as the Gulf of Mexico.

Several sediment trap studies from different latitudes have documented the coccolith variable fluxes, gaining some insights into the controls that modulate export production. For example, in the NE Atlantic (34^o^N 21^o^W and 48^o^N 21^o^W), Broerse et al. [13] found marked seasonal patterns with maximum coccolith fluxes during the regional spring bloom period dominated by *Emiliania huxleyi*, while in late autumn coccolith assemblage is dominated by *Florisphaera profunda*. These species accounted for 85% of the total coccolith fluxes in the Bermuda Station (N. Atlantic) in the subtropical gyre, characteristic for stable surface water stratification, low primary production, and low seasonality [9]. The mixing associated with mesoscale eddies can efficiently resupply nutrients to the surface waters and erode the stratification, leading to an increase in primary productivity and consequently to higher export of biogenic particle fluxes, including the coccolithophores, as observed in the sediment traps data [9,13]. On the other hand, in upwelling regions, such as the Cape Blanc off NW Africa, upper photic zone (UPZ) species dominated by *E. huxleyi* and *Gephyrocapsa oceanica* are most abundant under these mesotrophic conditions [14,15]. These abundances decline gradually towards the Caribbean, where higher sea surface temperatures lead to decreasing chlorophyll-a, and to an increase in importance of the lower photic zone (LPZ) species, such as *F. profunda* and *Gladiolithus flabellatus*, which are adapted to low light conditions, showing fluxes up to 3–5 times higher in the western Caribbean region. These species changes apparently follow the geostrophic wind-forced deepening of the thermocline/nutricline [16]. This steep gradient in the UPZ and LPZ species across the tropical North Atlantic is associated with a weak seasonality in coccolith fluxes, and the Western and central Tropical N. Atlantic sites are modulated by the latitudinal migration of the Intertropical Convergence Zone. In contrast to the Eastern tropical N. Atlantic, where the variability induced by the wind-forced Cape Blank upwelling system shows the strongest upwelling typically during late winter [17].

In the Gulf of Mexico (GoM), there are some coccolithophore studies in the water column, providing some valuable spatial information on the community structure and the synoptic oceanographic and climatic conditions that modulate them in the photic zone [18–24]. However, these studies do not provide insight into seasonal/temporal variability as do time series studies with sediment traps, which severely limits our knowledge of the seasonal to interannual variability of coccolithophore flux in this region and their controlling factors. Understanding these dynamics is essential for deciphering the environmental processes that influence coccolithophore production and their role in the global carbon cycle.

Here, we present data on coccolith fluxes and their seasonal variability from two sediment traps located in the Perdido and Coatzacoalcos regions, western and southwestern parts of the GoM, and use satellite remote sensing data and outputs from numerical models for the same period to explore environmental parameters that might affect their production and flux.

### Oceanographic conditions in the Gulf of Mexico

**Fig 1:**
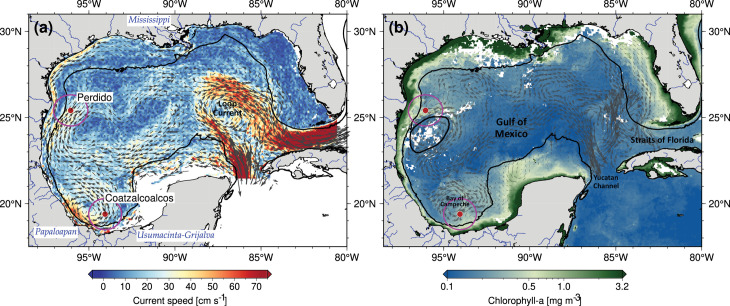
Study area in the southern Gulf of Mexico (GoM). (A) mean surface circulation patterns of the Gulf of Mexico based on data obtained from surface drifters in Lilly and Pérez-Brunius [42]. Vectors indicate the direction of the current, and the colors correspond to the current speed. Sediment trap locations at Perdido and Coatzacoalcos are indicated by red dots, highlighting the assumed area of influence caught by the sediment traps by the circles in magenta. Major rivers flowing into the Gulf of Mexico are further highlighted in the map. (B) Snapshot of the surface currents derived from satellite altimetry measurements (gray vectors) and the position of a large Loop Current Eddy called Poseidon (black contour) on January 1st, 2017. Blue-green colors represent the 9-day average chlorophyll-a field (December 28th, 2016, to January 5th, 2017). Chlorophyll-a was averaged to attenuate the effects of clouds. Trap locations and influence regions are shown. Note the filament with chlorophyll-a concentration around 0.5 mg m^-3^, extending by approximately 200 km along 25° N, from the 200 m isobath into deeper waters to the east. The filament was most probably produced by the interaction of the Loop Current Eddy with the region of high chlorophyll-a concentration (> 0.5 mg m^-3^) on the continental shelf.

The Gulf of Mexico, located between 18–30°N and 80–98°W (Fig 1), is a semi-closed ocean basin filled with different water masses from the Caribbean Sea by the strong Yucatan current. As this important western boundary current enters the GoM, it flows in a clockwise direction in the eastern region of the GoM and gives rise to the Loop Current that eventually exits at the GoM through the Straits of Florida. Sporadically, over a couple of months period, this Loop Current detaches from the main incoming flow, shedding a large anticyclonic eddy. These mesoscale anticyclonic eddies then move westward into the western GoM at speeds of 2–5 km d^-1^, interacting with other eddies in the GoM [25–28]. These transient events can last up to a year [29,30], and are the source of the physical and biogeochemical variability within the GoM, advecting salt, heat, carbon, nutrients, and oxygen into the interior of the GoM [31–33], as well as picophytoplankton populations as *Prochlorococcus* spp., and a diverse biota [34] towards the western region of the GoM. In the Bay of Campeche (southern region of the GoM), a semi-permanent cyclonic eddy [35] promotes Ekman pumping at its center, resulting in the shallowing of the nutricline, and is associated with the upwelling of nutrients into the euphotic zone [36]. The GoM basin can be divided into a euphotic coastal water region with high primary productivity controlled by circulation features on the shelf, in stark contrast with the offshore oligotrophic deep waters in the central part of the GoM characteristic for their low primary productivity values due to the stronger year around stratification, and consequently low nutrient concentration at the surface [37–41].

In the GoM deep water region, chlorophyll concentrations show seasonal variability related to the injection of nutrients into the euphotic zone and controlled by the deepening of the mixed layer deep (MLD) during winter. In contrast, strong stratification during spring-summer is characterized by low nutrient and chlorophyll concentrations in the photic zone [41,43]. However, open water cannot be considered a homogeneous region in regard to chlorophyll dynamics. Damien et al. [39] discussed the seasonal interannual variability of chlorophyll concentration in the GoM, using a coupled physical-biogeochemical model to analyze biogeochemical regions and nutrient dynamics, resulting in three regions. In this study, they describe Region 1 (southern part of GoM, including the Coatzacoalcos region) as showing no significant increase in total chlorophyll (Chl-t) content in winter, due to the relatively shallow MLD that does not reach the nutrient-rich subsurface waters, resulting in low winter nitrate concentrations and no significant increase in chlorophyll biomass. In Region 2 (central part of the GoM including the Perdido region), they report an Increase in Chl-t in winter only during specific years, showing substantial interannual variability influenced by atmospheric buoyancy fluxes and the interaction with Loop Current Eddies (LCEs). In Region 3 (northern part of the GoM, mainly on the Louisiana and Texas continental shelf), they report recurrent increases in Chl-t in winter, influenced by the Mississippi River discharges. The deepening of the MLD in winter is associated with the mixing of subsurface waters due to wind stress [44], to which enhanced continental water discharges from sixteen rivers are an important control of the increased nutrient availability in the continental shelf region [45]. In the northern part of the GoM, especially the Mississippi and the Atchafalaya rivers, significantly impact the marine ecosystem, particularly during the period of maximum water discharges in spring (up to 2.8 x 10^4^ m^3^ s^-1^). In the southern part of the GoM, the Grijalva-Usumacinta River complex, flowing through the Mexican state of Tabasco, and the Coatzacoalcos-Papaloapan rivers in Veracruz are major freshwater sources with maximum discharges in summer through early autumn, with an annual flux of 0.38 x 10^4^ m^3^ s^-1^. These river inputs supply nutrients and terrigenous sediments to the southern part of the Campeche Bay, which impacts the phytoplankton growth dynamics in this region [40,46,47].

## Materials and methods

### Trap deployment and sample treatment

Two sediment traps were deployed offshore the Perdido and Coatzacoalcos region in the western and southern Gulf of Mexico, collecting sinking particles from June 2016 to July 2017, located at 1100 m and 1050 m, respectively, with a sampling interval resolution of 18 days (Fig 1, Table 1). At the time of the trap deployment (named XIXIMI cruises), the pertinent marine authorities, SEMAR (https://www.gob.mx/semar) and SEMARNAT (https://www.gob.mx/semarnat), were notified. For greater transparency in the research, we included a complete copy of PLOS’s questionnaire on inclusivity in global research in the supplementary material (S1 File).

**Table 1:** Sediment trap mooring information deployed in the Gulf of Mexico. There is a delay between the Perdido and Coatzacoalcos trap timing because it was not possible to install the sediment trap due to the passage of Tropical Storm Danielle (Jun 19 to Jun 21, 2016). Both traps were sampled for 378 days. Sample lost during sediment trap recovery (*).

Location	Perdido trap		Location	Coatzacoalcos trap	
	25.43111° N and 96.07207° W			19.3867° N and 94.0597° W	
Collection bottle	Begin date	End date	Collection bottle	Begin date	End date
			1	16/06/2016	03/07/2016
1*	04/07/2016	21/07/2016	2	04/07/2016	21/07/2016
2	22/07/2016	08/08/2016	3	22/07/2016	08/08/2016
3	09/08/2016	26/08/2016	4	09/08/2016	26/08/2016
4	27/08/2016	13/09/2016	5	27/08/2016	13/09/2016
5	14/09/2016	01/10/2016	6	14/09/2016	01/10/2016
6	02/10/2016	19/10/2016	7	02/10/2016	19/10/2016
7	20/10/2016	06/11/2016	8	20/10/2016	06/11/2016
8	07/11/2016	24/11/2016	9	07/11/2016	24/11/2016
9	25/11/2016	12/12/2016	10	25/11/2016	12/12/2016
10	13/12/2016	30/12/2016	11	13/12/2016	30/12/2016
11	31/12/2016	17/01/2017	12	31/12/2016	17/01/2017
12	18/01/2017	04/02/2017	13	18/01/2017	04/02/2017
13	05/02/2017	22/02/2017	14	05/02/2017	22/02/2017
14	23/02/2017	12/03/2017	15	23/02/2017	12/03/2017
15	13/03/2017	30/03/2017	16	13/03/2017	30/03/2017
16	31/03/2017	17/04/2017	17	31/03/2017	17/04/2017
17	18/04/2017	05/05/2017	18	18/04/2017	05/05/2017
18	06/05/2017	23/05/2017	19	06/05/2017	23/05/2017
19	24/05/2017	10/06/2017	20	24/05/2017	10/06/2017
20	11/06/2017	28/06/2017	21	11/06/2017	28/06/2017
21	29/06/2017	16/07/2017			

Each mooring was equipped with a McLane automated time-series sediment trap, model Par flux Mark 78H-21 with a 0.5 m^-2^ collection opening cone, and twenty-one collection bottles of 500 ml capacity. Each collection bottle was prefilled with a preserving 4% formaldehyde water solution to minimize biological decomposition, filtered (0.45 µm) seawater, buffered with sodium tetraborate and sodium chloride (both high purity) to 8.5 pH to minimize the dissolution of carbonates [48].

Collection bottles were sieved through a 1000 µm Nylon screen to remove “swimmers” [49,50]. After sieving, each collection bottle was divided into ten equal subsamples (50 ml each) using a wet sampler divider (WSD-10 McLane Laboratory) with deviations between subsamples <5.0% [51].

### Coccolith analysis

The sediment trap samples were further wet split into smaller aliquots employing a Sampler Rotatory Divider Laborette 27 (we used the split of 2/60). We calculated the standard error by analyzing four samples in triplicate (3, 10, 14, and 21 for each sample set), resulting in a 0.4% error between splits. After fecal pellets were disintegrated and the organic matter was removed, with two strong oxidants, sodium hypochloride (NaClO) and hydrogen peroxide (H_2_O_2_), the aggregates were mechanically disintegrated by repeated short periods of ultrasonification (50 kHz for 5 s) [52], samples were filtered onto Nucleopore membranes (47 mm diameter, 0.45 μm pore size). A piece of the membrane filter was mounted on aluminum stubs and sputtered with 3 nm of platinum using a BalTec SCD500 sputter for subsequent automated acquisition of 500 images per sample at 1500x magnification on a scanning electron microscope (Zeiss Supra 55VP) [53,54]. The total area covered by the 500 images is 2.13 x 10^−6^ m^-2^ per sample.

The image analysis software Image-J for Windows® was used to count (custom plugin) and measure the size of coccoliths, aiding in taxonomic classification. The general identification of coccoliths includes the identification of morphotypes of *Emiliania huxleyi* type A (*huxleyi*), type B (*pujosiae*), and *E. huxleyi* var. *corona*., followed by the taxonomic concept described by Young et al. [55], and the Nannotax web site (https://www.mikrotax.org/Nannotax3/index.html Accessed 21 Apr. 2022). Morphotypes of *Florisphaera profunda* were identified according to Quinn et al. [56], morphotypes of *Gephyrocapsa oceanica*, *G. muellerae*, and *G. ericsonii* were identified according to Bollmann [57], and species within the *Syracosphaera* group were identified according to Kleijne and Cros [58]. All coccoliths were identified at the species level except for some specimens of *Syracosphaera*, which are reported as spp. Taxa with an average relative abundance larger than 2% are referred to as “most common taxa” while species with relative abundances less than 2% are referred to as “Other coccoliths” in the entire manuscript.

Additionally, coccolith fragments in groups (ranging from 25 to 75% visual inspection) were quantified and included as complete species. The coccoliths reconstructed as complete from their fragments accounted for less than 2% of the total coccoliths per sample per species. The coccolith flux (coccoliths per m^-2^ d^-1^) was calculated following Andruleit [59].

Eq. (1):


$$Cf=\frac{N*A*Sf}{a*t*Mt}$$ (1)

where Cf = Coccolith flux; N = number of coccoliths; A = Effective filtered area (9.89 x 10^-04^ m^-2^); Sf = Split factor (4500); a = analyzed filter area (2.13x10^-06^ m^-2^); t = sediment collection time (18 days), and Mt = Mouth of sediment trap (0.5).

The relative abundance for each coccolith species in each sample was calculated as the following equation:


$$RA=\frac{ni}{N}*100$$ (2)

Where *RA* = Relative abundance (%), *ni* = number of coccoliths of the species *i*, and *N* = Total number of coccoliths in the sample.

### Ecological indices

We used the number of species observed in the sample as the species richness “*s*”, for the Diversity Index, we used Shannon and Weaver [60]; calculated with the following equation:


$$H´=-\sum_{i=1}^{s}Pi*lnPi$$ (3)

Where: *H*´ = diversity; *S* = the number of species; Pi = relative or proportional abundance of *i* species (equal to ni/N), where ni is the number of coccoliths by species and N is the total number of coccoliths. For the Evenness we used Pielou [61] with the following equation:


$$J=\frac{H^{\prime }}{ln(s)}$$ (4)

Where: *H*’ is Shannon-Weaver’s diversity and (*s*) the number of species observed in the sample. Finally, the Dominance Index Berger and Parker [62] “d” with the following equation:


$$d=\frac{nmax}{N}$$ (5)

Where: *d* = dominance index; *nmax* = the number of individuals of the most abundant species, and *N* = the total number of individuals in the sample.

We grouped the coccolithophore assemblages into the upper photic zone (UPZ) and lower photic zone (LPZ) based on their reported ecological preferences from plankton studies [63–67]. The ratio between the UPZ/LPZ taxa was used to identify variations of the nutricline [17,66,68] since the boundary between LPZ and UPZ is approximately the position of the deep chlorophyll maximum, nutricline, and Photosynthetic Available Radiation at a level of 1% [2]. All the coccolith datasets used in this work are indicated in S1 Table.

### Remote sensing data

Surface oceanographic data from remote sensing and numerical models were retrieved within a one-degree radius around each sediment trap location. This area potentially includes the source regions of sinking aggregates and marine phytoplankton reaching the sediment trap at 1100 m water depth (e.g., [69]), referred to in this work as “the assumed area of influence caught by the sediment trap” (see Fig 1). Surface data were additionally averaged over the 18-day collecting period of each sediment trap cup to relate the oceanographic surface variability with the sediment trap data. The surface time series were also used on the ordination of the multivariate analysis described in the next section. Data sources and more detailed information are given in the supplementary material in S2 Table.

Sea Surface Temperature (SST) was used as a proxy for thermal stratification [70]. Sea surface chlorophyll a (Chl-a) was used as an indicator of primary productivity variability [71]. To determine the presence of anticyclonic eddies within the radius of the assumed area of influence caught by the sediment traps, we used 0.20–0.25 m amplitudes of sea level anomaly (SLA) as described by Huang et al. [72], data from the Copernicus Marine Service (CMEMS; https://doi.org/10.48670/moi-00148). Wind Speed (WS) data [73,74] allow us to identify the occurrence of intense wind bursts known as the “Nortes” related to outbreaks of cold air from the North American continent during late autumn and winter. These winds provide mechanical energy to the sea surface and control the Mixed Layer Depth (MLD) [75] and consequently nutrient availability and Photosynthetic Available Radiation (PAR) [76]. Precipitation (PREC) can provide information for the general understanding of weather patterns and seasonality [77] and, indirectly, the freshwater influx from rivers into the Perdido and Coatzacoalcos region.

### Statistical analysis

Principal Components Analysis (PCA) was carried out with environmental parameters based on a correlation matrix within groups. Environmental parameters were standardized by subtracting the mean and dividing by the standard deviation [78]. The relationship between the flux of coccoliths and environmental parameters during the study period was explored with the aid of a Spearman rank correlation and Canonical Correspondence Analysis. These analyses provide an integrated description of the species-environment relationship, allowing for the identification of the environmental factors that best explain the variation in the assemblage composition [79]. We used the software package Past 4.03. The data matrix included Mixed Layer Deep (MLD), Sea Level Anomaly (SLA), Geostrophic Speed (GOS), Wind Speed (WS), Precipitation, Photosynthetically Active Radiation (PAR), Sea Surface Temperature (SST), Sea Surface Salinity (SSS), Chlorophyll a (Chl-a) and the coccolith fluxes.

## Results

### Oceanographic and meteorological conditions for the Perdido and Coatzacoalcos area during the study period

**Fig 2:**
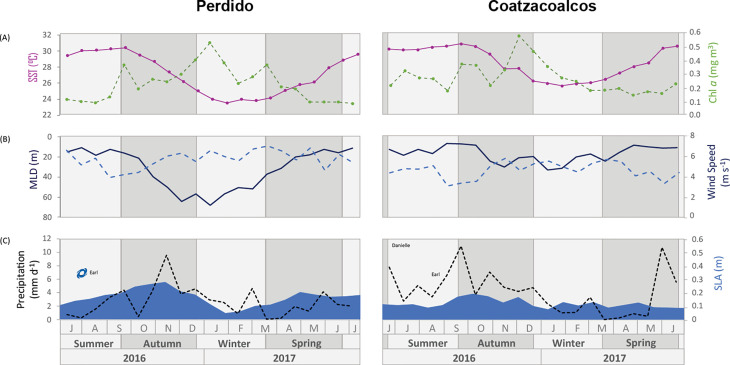
Environmental conditions at the Perdido and Coatzacoalcos areas during the study period. (A) Sea Surface Temperature (magenta line with solid circles) and Chlorophyll a (green broken line). (B) Mixed Layer Depth (dark blue line) and wind speed (blue broken line). (C) Precipitation (black broken line) and Sea Level Anomaly (blue background). Tropical Storm Danielle and Hurricane Earl (category 1), which mainly affected the Coatzacoalcos area, are highlighted. Light and dark grey bands mark the seasons of the year.

Sea surface variability captured in the Sea Surface Temperatures (SSTs), MLD, and Chl-a shows similar seasonal behavior between the two sediment trap locations, though with different absolute values and ranges. SSTs reach similar maximum values in both regions during summer, while during winter, the Perdido region cools slightly more than Coatzacoalcos (Fig 2A, B). Chl-a shows the opposite pattern, higher values during sea surface cooling in autumn and winter, and lower values during the warmer months. The MLD is consistently deeper during cooling in late autumn until winter in both areas; however, it is deeper in the Perdido than in Coatzacoalcos (68 and 33 m, respectively). Wind speeds are slightly more intense in the Perdido area than in Coatzacoalcos, especially during winter, reaching 7.0 m s^-1^ and 5.9 m s^-1^, respectively.

Record of precipitation in Coatzacoalcos shows two maxima; the first in late summer (max 10.9 mm d^-1^) and the other in late spring 2017 that reached 10.7 mm d^-1^, due to the passage of tropical storm Danielle (June 19 to June 21 of 2016), and category one hurricane Earl (2 to August 06 of 2016). Precipitation in Perdido shows relatively lower values with a maximum of 9.5 mm d^-1^ during autumn (https://www.nhc.noaa.gov/data/tcr/index.php?season=2016&basin=atl).

SLA shows noticeable differences between the Perdido and Coatzacoalcos, especially in their time variability; Perdido SLA follows more closely the annual cooling and warming cycle, in contrast to Coatzacoalcos, which shows a much higher frequency most probably due to the passage of mesoscale eddies and shelf to offshore currents in this region (https://www.horizonmarine.com/loop-current-eddies), (Fig 2C).

### Coccolithophore species diversity and composition

A total of 41 samples were recovered throughout the study period: 20 for the Perdido trap (sample 1 was lost: 07/04/2016-07/21/2016) and 21 for the Coatzacoalcos trap. We identified 47 species in the Perdido trap samples, and 56 in the Coatzacoalcos (Table 2). The metrics of diversity Indices, species richness (*s*), Diversity (Shannon-Weaver, *H*’), and evenness (Pielou, *J*) are inversely proportional to the dominance Index (Berger-Parker, *d*) and show different seasonal patterns throughout the study period in each trap locations.

**Table 2:** Taxonomic list of coccolithophore species recorded for the Perdido and Coatzacoalcos traps. The scientific name of the taxa, Bibliographic references, and location: Perdido = P and Coatzacoalcos = C, as well as coccolith type: Heterococcolith = HET, Holococcolith = HOL, Nannolith = NAN. Dinoflagellate = DIN (Thoracosphaera spp. Was the only one recorded but not included in the Total coccolith fluxes).

TAXA	Bibliographic references	Location	Life-Cycle
*Acanthoica acanthifera*	Lohmann 1912 ex Lohmann, 1913	P-C	HET
*Acanthoica acanthos*	Schiller 1925	C	HET
*Acanthoica quattrospina*	Lohmann 1903	P-C	HET
*Acanthoica sp.*	Lohmann 1903	P	HET
*Algirosphaera robusta*	(Lohmann 1902) Norris, 1984	P-C	HET
*Braarudosphaera bigelowi*	(Gran & Braarud 1935) Deflandre, 1947	P-C	NAN
*Calcidiscus leptoporus small*	(Murray & Blackman 1898) Loeblich & Tappan, 1978	P-C	HET
*Calciosolenia brasiliensis*	(Lohmann, 1919) Young in Young et al., 2003	P-C	HET
*Calciosolenia murrayi*	Gran, 1912	P-C	HET
*Calyptrosphaera oblonga*	Lohmann 1902	C	HOL
*Ceratolithus cristatus*	Kamptner 1950	P-C	HET
*Ceratolitus cristatus*	(Kamptner 1950) CER cristatus type sensu Young et al., 2003	P-C	NAN
*Coccolithus pelagicus*	(Wallich 1877) Schiller, 1930	P-C	HET
*Coronosphaera mediterranea*	(Lohmann 1902) Gaarder, in Gaarder & Heimdal, 1977 [according to Triantaphyllou et al. 2016 and Young et al. 2020]	P-C	HET
*Cyrtosphaera aculeata*	(Kamptner 1941) Kleijne, 1992	P-C	HET
*Cyrtosphaera cidaris*	(Schlauder 1945) Young & Bown 2014	P-C	HET
*Discosphaera tubifera*	(Murray & Blackman 1898) Ostenfeld, 1900	P-C	HET
*Emiliania huxleyi type A*	(Lohmann 1902) Hay & Mohler, in Hay et al. 1967	P-C	HET
*Emiliania huxleyi type B*	Young & Westbroek, 1991	P-C	HET
*Emiliania huxleyi var. Corona*	(Okada & McIntyre 1977) Jordan & Young, 1990	P	HET
*F. profunda var. elongata*	Okada & MacInyre 1980	P-C	NAN
*F.profunda var. rhinocera*	Quinn et al., 2005	P-C	NAN
*Florisphaera profunda*	Okada & Honjo, 1973	P-C	NAN
*Gephyrocapsa ericsonii*	(McIntyre & Bé, 1967)	P-C	HET
*Gephyrocapsa mullerae*	Bréhéret, 1978	P-C	HET
*Gephyrocapsa oceanica*	Kamptner, 1943	P-C	HET
*Gladiolithus flabellatus*	(Halldal & Markali 1955) Jordan & Chamberlain, 1993	P-C	NAN
*Hayaster perplexus*	(Bramlette & Riedel 1954) Bukry, 1973	P-C	HET
*Helicosphaera carteri*	(Wallich 1877) Kamptner, 1954	P-C	HET
*Helicosphaera pavimentum*	Okada & McIntyre, 1977	P-C	HET
*Helicosphaera wallichii*	(Lohmann 1902) Okada & McIntyre, 1977	P-C	HET
*Michaelsarsia elegans*	Gran, 1912	P-C	HET
*Oolitothus antillarum*	(Cohen 1964) Reinhardt, in Cohen & Reinhardt, 1968	C	HET
*Oolitothus fragilis*	(Lohmann 1912) Martini & Müller, 1972	P-C	HET
*Ophiaster formosus*	Gran, 1912	P-C	HET
*Ophiaster hydroideus*	(Lohmann 1903) Lohmann, 1913	P-C	HET
*Ophiaster reductus*	Manton & Oates, 1983	C	HET
*Palusphaera vandelli*	Lecal, 1965	P-C	HET
*Papposphaera spp.*	Tangen, 1972	P	HET
*Ponthosphaera spp.*	Lohmann, 1902	P-C	HET
*Pontosphaera multipora*	(Kamptner, 1948 ex Deflandre in Deflandre & Fert, 1954) Roth, 1970	P-C	HET
*Poricalyptra spp.*	Kleijne 1991	P-C	HOL
*Reticulofenestra parvula*	(Okada & McIntyre 1977) Bendif, Probert, Young & von Dassow in Bendif et al. 2016	C	HET
*Reticulofenestra sessilis*	(Lohmann 1912) Jordan & Young, 1990	P-C	HET
*Reticulofenestra sp.*	Hay, Mohler & Wade, 1966	P-C	HET
*Rhabdosphaera clavigera var. stilifera*	(Lohmann, 1902) Kleijne&Jordan, 1990	P-C	HET
*Scyphosphaera apsteinii*	Lohmann, 1902	P-C	HET
*Solisphaera blagnasensis*	Bollmann et al., 2006	P-C	HET
*Syracolithus quadriperforatus*	(Kamptner 1937) Gaarder, in Heimdal & Gaarder 1980	P-C	HOL
*Syracosphaera corolla*	Lecal, 1966 emend Young et al. 2018	C	HET
*Syracosphaera gaarderae*	(Okada & McIntyre 1977) Keuter, Young & Frada 2019	P-C	HET
*Syracosphaera histrica*	Kamptner, 1941	C	HET
*Syracosphaera lamina*	Lecal-Schlauder 1951	P-C	HET
*Syracosphaera molischii*	Schiller 1925	P-C	HET
*Syracosphaera nana*	(Kamptner 1941) Okada & McIntyre, 1977	P-C	HET
*Syracosphaera nodosa*	Kamptner, 1941	P-C	HET
*Syracosphaera noroitica*	Knappertsbusch, 1993	C	HET
*Syracosphaera orbiculus*	Okada & McIntyre, 1977	C	HET
*Syracosphaera ossa*	(Lecal 1966) Loeblich & Tappan, 1968	C	HET
*Syracosphaera pulchra*	Lohmann, 1902	P-C	HET
*Syracosphaera rotula*	Okada & McIntyre, 1977	P-C	HET
*Syracosphaera tumularis*	Sánchez-Suárez, 1990	P-C	HET
*Umbellosphaera irregularis*	Paasche, in Markali & Paasche 1955	P-C	HET
*Umbellosphaera tenuis*	Kamptner, 1937 Paasche, in Markali & Paasche 1955 emend. Paasche 1955	P-C	HET
*Umbicosphaera hulburtiana*	Gaarder 1970	P-C	HET
*Umbilicosphaera foliosa*	(Kamptner 1963) Geisen in Sáez et al., 2003	P-C	HET
*Umbilicosphaera sibogae*	(Weber – van Bosse 1901) Gaarder, 1970	P-C	HET
*Zygosphaera hellenica*	Kamptner 1937	P-C	HOL
*Thoracosphaera spp.*	(Lohmann 1920) Kamptner 1944	P-C	DIN

**Fig 3:**
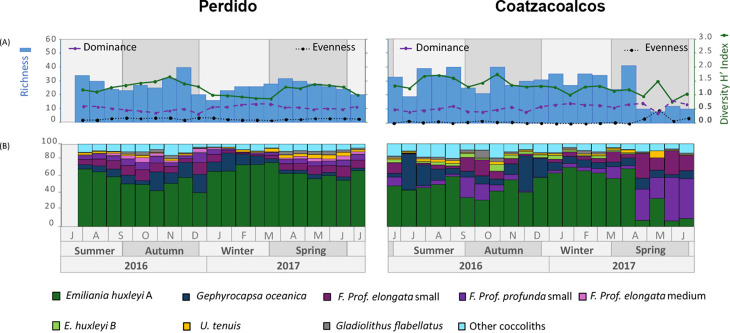
Ecological indices to the Perdido and Coatzacoalcos traps. (A) shows the Species richness (blue bars), Shannon-Weaver Diversity Index (green line with dots), Evenness (black dotted line), and Berger-Parker Dominance Index (purple color). The y-axis on the right shows Shannon-Weaver (0-3), Evenness, and Dominance (0-1 bold). (B) The relative abundance (%) of the taxa for each sample. Taxa with lower relative abundances (<2%) are grouped in “Other coccoliths”.

In the Perdido trap, the species richness ranges from 16 in late winter to 40 species in late autumn; the Shannon-Weaver Diversity Index ranges from 0.8 in late winter to 1.6 in mid-autumn, and the evenness record changes little throughout the year. On the other hand, the Coatzacoalcos trap shows some variability throughout the study period and a decrease in diversity Indices in spring 2017. Species richness ranged from a maximum of 41 in early spring to a lowest of 8 in mid-spring of 2017. The Shannon-Weaver diversity Index ranges from 0.8 in late spring of 2017 to 1.7 in mid-autumn, and the evenness ranged from 0.08 in early spring to 0.5 in mid-spring. In general, the Berger-Parker Dominance Index was higher in winter (Fig 3A). These trends in the Diversity and Dominance Indices are further reflected in the relative abundance of each species.

Of all the taxa recorded in the sediment traps, only 7 showed an average relative abundance greater than 2% during the study period. These taxa are referred to as the “most common taxa” in this study. *Emiliania huxleyi* type A was the dominant species, averaging 61% throughout the study period in the Perdido trap and 46% in the Coatzacoalcos trap, showing higher values in winter in both traps. *Gephyrocapsa oceanica* shows higher relative abundances in autumn and winter, meaning 10% in the Perdido trap and 13% in the Coatzacoalcos trap, while this last region shows higher values in mid-summer and late autumn. In general, *Florisphaera profunda* shows higher values in autumn and spring in both traps. *F. profunda* var. *elongata* (small), with mean relative abundances of 8% in Perdido and 10% in Coatzacoalcos, and *F. profunda* var. *profunda* (small), averaging 5% in Perdido and 13% in Coatzacoalcos, show higher values during spring 2017 in the Coatzacoalcos trap. Relatively lower abundances of *F. profunda* var. *elongata* (medium) range from 3% in Perdido and 1% in Coatzacoalcos. On the other hand, *Umbellosphaera tenuis* and *Gladiolithus flabellatus* show means around 2% throughout the study period in both traps. *E. huxleyi* type B shows mean values of 2% in the Perdido and in Coatzacoalcos, while the group “Other coccoliths” in the Perdido showed maximum values of 8% and 10% in the Coatzacoalcos trap. A comparison of the temporal variation of relative abundances is shown in Fig 3B, while the temporal variation of each taxon separately can be seen in Fig 5 (right axis).

**Fig 4:**
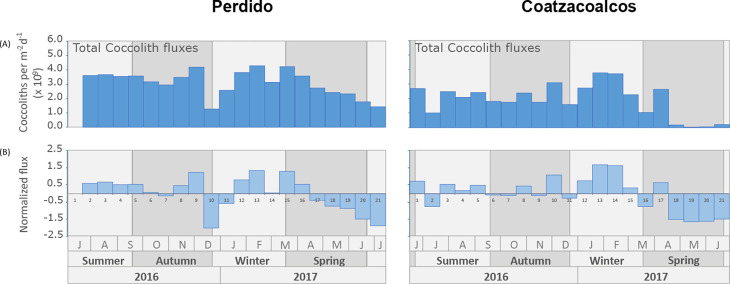
Seasonality of total coccolith fluxes and Normalized fluxes. (A) Total coccolith fluxes (coccoliths per m^-2^ d^-1^ x 10^9^; blue bars) and (B) the normalized flux, deviation from the annual mean coccolith flux, with the sample number for the Perdido and Coatzacoalcos traps.

**Table 3:** Relative abundances and coccolith fluxes (x 10^9^) for the Perdido and Coatzacoalcos traps. Range, average, and standard deviation for each case. Relative abundance > 2% on average throughout the study period was considered abundant coccolithophore taxa (loadings marked and *), while relative abundances < 2% were grouped in Other coccoliths. Relative abundances < 0.05 on average are shown to three decimal places.

Taxa	The Perdido Trap							
	Relative abundances (%)				Fluxes of coccoliths per m^-2^ d^-1^ (x 10^9^)			
	Min	Max	Average	StDev	Min	Max	Average	StDev
Total flux coccoliths					1.300	4.292	3.099	0.891
E. huxleyi type A*	40.9	77.2	61.3	10.0	0.532	3.276	1.934	0.725
G. oceanica*	3.3	23.2	10.2	6.7	0.048	0.849	0.324	0.235
F. profunda var. elongata small*	1.6	14.3	8.1	3.7	0.060	0.412	0.230	0.098
F. profunda var. profunda small*	0.8	11.6	5.4	2.6	0.033	0.290	0.154	0.067
F. profunda var. elongata medium*	0.4	6.0	2.7	1.6	0.019	0.189	0.075	0.043
U. tenuis*	0.4	5.1	2.3	1.3	0.005	0.152	0.071	0.038
G. flabellatus*	0.2	4.5	2.1	1.6	0.006	0.153	0.060	0.050
*Others coccoliths*	3.4	14.2	7.8	2.7	0.049	0.498	0.251	0.122
*E. huxleyi type B*	0.2	4.3	1.9	1.0	0.003	0.136	0.060	0.037
*U. irregularis*	0.3	3.9	1.5	0.9	0.004	0.135	0.049	0.032
*U. sibogae*	0.3	2.1	0.9	0.5	0.005	0.077	0.029	0.020
*S. pulchra*	0.0	1.3	0.7	0.4	0.000	0.048	0.021	0.014
*F. profunda var. elongata large*	0.1	0.9	0.4	0.3	0.001	0.029	0.012	0.007
*H. carteri*	0.0	0.5	0.3	0.1	0.000	0.019	0.009	0.006
*F. profunda var. profunda medium*	0.0	0.5	0.3	0.1	0.001	0.014	0.007	0.003
*C. leptoporus*	0.1	0.5	0.2	0.1	0.001	0.020	0.006	0.004
*C. brasiliensis*	0.0	1.0	0.2	0.3	0.000	0.034	0.006	0.010
*C. cristatus HET*	0.0	0.4	0.2	0.1	0.001	0.014	0.005	0.004
*U. hulburtiana*	0.0	0.3	0.1	0.1	0.000	0.010	0.005	0.003
*G. ericsonii*	0.0	0.3	0.1	0.1	0.000	0.011	0.005	0.003
*R. sessilis*	0.0	0.3	0.1	0.1	0.000	0.010	0.004	0.002
*G. mullerae*	0.0	0.3	0.1	0.1	0.000	0.010	0.004	0.003
*U. foliosa*	0.0	0.5	0.1	0.1	0.000	0.022	0.003	0.005
*F. profunda var. rhinocera*	0.0	0.3	0.1	0.1	0.000	0.007	0.003	0.002
*Reticulofenestra sp.*	0.0	0.5	0.1	0.1	0.000	0.023	0.003	0.005
*S. molischii*	0.0	0.3	0.1	0.1	0.000	0.011	0.003	0.003
*S. lamina*	0.0	0.4	0.1	0.1	0.000	0.013	0.002	0.004
*R. clavigera*	0.000	0.403	0.060	0.122	0.00000	0.01694	0.00220	0.00474
*C. murrayi*	0.000	0.213	0.048	0.064	0.00000	0.00673	0.00161	0.00227
*H. perplexus*	0.000	0.161	0.040	0.051	0.00000	0.00534	0.00135	0.00179
*H. wallichii*	0.000	0.128	0.040	0.039	0.00000	0.00534	0.00131	0.00143
*S. nana*	0.000	0.202	0.039	0.058	0.00000	0.00706	0.00135	0.00206
*D. tubifera*	0.000	0.370	0.029	0.084	0.00000	0.01555	0.00113	0.00350
*C. pequeña*	0.000	0.107	0.029	0.030	0.00000	0.00259	0.00085	0.00080
*C. Cristatus CER*	0.000	0.157	0.028	0.037	0.00000	0.00466	0.00085	0.00115
*R. clavigera var. stylifera*	0.000	0.149	0.018	0.039	0.00000	0.00627	0.00069	0.00157
*O. antillarum*	0.000	0.059	0.016	0.016	0.00000	0.00213	0.00051	0.00056
*M. elegans*	0.000	0.072	0.015	0.024	0.00000	0.00260	0.00046	0.00074
*O. formosus*	0.000	0.236	0.014	0.053	0.00000	0.00846	0.00053	0.00192
*S. apsteinii*	0.000	0.098	0.014	0.025	0.00000	0.00352	0.00049	0.00087
*P. multipora*	0.000	0.031	0.010	0.010	0.00000	0.00116	0.00036	0.00037
*A. quattrospina*	0.000	0.065	0.009	0.017	0.00000	0.00280	0.00036	0.00070
*C. mediterranea*	0.000	0.039	0.007	0.012	0.00000	0.00142	0.00023	0.00042
*Poricalyptra sp.*	0.000	0.033	0.007	0.010	0.00000	0.00118	0.00023	0.00036
*P. vandelli*	0.000	0.053	0.006	0.014	0.00000	0.00070	0.00013	0.00025
*S. blagnasensis*	0.000	0.052	0.003	0.012	0.00000	0.00092	0.00007	0.00021
*C. cidaris*	0.000	0.027	0.003	0.006	0.00000	0.00094	0.00011	0.00022
*S. rotula*	0.000	0.013	0.002	0.004	0.00000	0.00047	0.00006	0.00013
*S. tumularis*	0.000	0.026	0.001	0.006	0.00000	0.00095	0.00005	0.00021
*O. hydroideus*	0.000	0.022	0.001	0.005	0.00000	0.00093	0.00005	0.00021
*S. gaarderae*	0.000	0.017	0.001	0.004	0.00000	0.00070	0.00003	0.00016
*C. pelagicus*	0.000	0.013	0.001	0.003	0.00000	0.00047	0.00002	0.00011
*C. aculeata*	0.000	0.011	0.001	0.002	0.00000	0.00046	0.00002	0.00010
*F. profunda var. elongata XL*	0.000	0.010	0.000	0.002	0.00000	0.00023	0.00001	0.00005
*A. robusta*	0.000	0.008	0.000	0.002	0.00000	0.00023	0.00001	0.00005
*S. quadriperforatus*	0.000	0.007	0.000	0.001	0.00000	0.00023	0.00001	0.00005
*A. acanthifera*	0.000	0.006	0.000	0.001	0.00000	0.00023	0.00001	0.00005
*B. bigelowi*	0.000	0.006	0.000	0.001	0.00000	0.00023	0.00001	0.00005
*S. nodosa*	0.000	0.006	0.000	0.001	0.00000	0.00023	0.00001	0.00005
*E. huxleyi var. corona*	0.000	0.005	0.000	0.001	0.00000	0.00023	0.00001	0.00005
*H. pavimentum*	0.000	0.005	0.000	0.001	0.00000	0.00023	0.00001	0.00005
*Thoracosphaera spp.*	0.000	0.031	0.014	0.011	0.00000	0.00116	0.00047	0.00040
Total flux coccoliths					0.014	3.785	1.887	1.132
E. huxleyi type A *	6.5	71.5	46.1	19.9	0.004	2.705	1.035	0.767
F. profunda var. profunda small *	0.6	52.7	13.5	15.8	0.003	0.445	0.128	0.114
G. oceanica *	3.3	43.5	13.3	11.3	0.001	1.345	0.259	0.294
F. profunda var. elongata small *	0.8	30.0	10.0	8.8	0.002	0.357	0.125	0.108
E. huxleyi type B *	0.0	8.7	2.4	2.2	0.000	0.208	0.057	0.051
U. tenuis *	0.4	8.5	2.4	1.8	0.000	0.093	0.043	0.032
G. flabellatus *	0.0	9.3	2.1	2.0	0.000	0.163	0.044	0.044
*Others coccoliths*	4.5	17.9	10.2	4.1	0.001	0.434	0.198	0.144
*U. sibogae*	0.0	3.9	1.5	1.2	0.000	0.097	0.032	0.029
*F. profunda var. elongata medium*	0.0	5.2	1.3	1.3	0.000	0.143	0.023	0.030
*U. irregularis*	0.0	3.1	1.1	0.9	0.000	0.078	0.022	0.021
*S. pulchra*	0.0	2.7	0.9	0.8	0.000	0.064	0.022	0.020
*C. cristatus HET*	0.0	7.2	0.8	1.7	0.000	0.018	0.006	0.005
*H. carteri*	0.0	1.1	0.5	0.3	0.000	0.026	0.011	0.008
*C. brasiliensis*	0.0	1.7	0.5	0.5	0.000	0.051	0.011	0.014
*F. profunda var. profunda medium*	0.0	1.4	0.4	0.4	0.000	0.039	0.008	0.010
*C. leptoporus small*	0.0	3.4	0.3	0.7	0.000	0.012	0.003	0.004
*Discosphaera tubifera*	0.0	1.6	0.3	0.4	0.000	0.038	0.007	0.011
*F. profunda var. elongata large*	0.0	1.5	0.3	0.3	0.000	0.042	0.007	0.009
*R. clavigera var. clavigera*	0.0	0.7	0.2	0.3	0.000	0.017	0.006	0.006
*C. leptoporus*	0.0	0.8	0.2	0.2	0.000	0.020	0.004	0.005
*S. tumularis*	0.0	1.7	0.2	0.4	0.000	0.013	0.003	0.004
*R. sessilis*	0.0	0.6	0.2	0.2	0.000	0.011	0.003	0.003
*O. antillarum*	0.0	1.7	0.2	0.4	0.000	0.006	0.002	0.002
*S. nana*	0.0	0.9	0.1	0.2	0.000	0.022	0.003	0.005
*F. profunda var. rhinocera*	0.0	0.6	0.1	0.1	0.000	0.008	0.002	0.002
*R. clavigera var. stylifera*	0.0	0.4	0.1	0.1	0.000	0.014	0.002	0.003
*U. hulburtiana*	0.0	0.3	0.1	0.1	0.000	0.008	0.002	0.002
*Poricalyptra sp.*	0.0	0.4	0.1	0.1	0.000	0.008	0.001	0.002
*S. molischii*	0.0000	0.1952	0.0594	0.0645	0.0000	0.0044	0.0014	0.0016
*C. cristatus CER*	0.0000	0.4082	0.0563	0.0919	0.0000	0.0030	0.0008	0.0009
*G. ericsonii*	0.0000	0.3287	0.0552	0.0741	0.0000	0.0090	0.0014	0.0020
*H. perplexus*	0.0000	0.2874	0.0551	0.0702	0.0000	0.0070	0.0013	0.0017
*O. formosus*	0.0000	0.4326	0.0521	0.1267	0.0000	0.0118	0.0009	0.0026
*C. cidaris*	0.0000	0.4790	0.0519	0.1250	0.0000	0.0116	0.0013	0.0031
*C. murrayi*	0.0000	0.2762	0.0483	0.0729	0.0000	0.0066	0.0013	0.0019
*G. mullerae*	0.0000	0.2105	0.0473	0.0609	0.0000	0.0050	0.0010	0.0012
*C. pelagicus*	0.0000	0.4082	0.0455	0.0897	0.0000	0.0021	0.0006	0.0007
*H. wallichii*	0.0000	0.2020	0.0442	0.0497	0.0000	0.0042	0.0011	0.0012
*C. mediterranea*	0.0000	0.2778	0.0421	0.0700	0.0000	0.0067	0.0011	0.0020
*A. robusta*	0.0000	0.2020	0.0393	0.0599	0.0000	0.0042	0.0009	0.0014
*Syracosphaera spp.*	0.0000	0.6891	0.0361	0.1500	0.0000	0.0172	0.0009	0.0037
*S. apsteinii*	0.0000	0.1170	0.0279	0.0351	0.0000	0.0021	0.0006	0.0007
*S. lamina*	0.0000	0.3802	0.0251	0.0858	0.0000	0.0086	0.0006	0.0020
*A. quattrospina*	0.0000	0.2142	0.0217	0.0489	0.0000	0.0053	0.0005	0.0012
*S. rotula*	0.0000	0.0958	0.0210	0.0280	0.0000	0.0023	0.0005	0.0007
*P. vandelli*	0.0000	0.0862	0.0167	0.0285	0.0000	0.0021	0.0004	0.0007
*M. elegans*	0.0000	0.1747	0.0166	0.0476	0.0000	0.0039	0.0004	0.0011
*P. multipora*	0.0000	0.0685	0.0149	0.0205	0.0000	0.0019	0.0003	0.0005
*U. foliosa*	0.0000	0.0931	0.0126	0.0282	0.0000	0.0023	0.0003	0.0007
*Reticulofenestra sp.*	0.0000	0.1130	0.0123	0.0288	0.0000	0.0025	0.0003	0.0007
*Acanthoica spp.*	0.0000	0.0898	0.0069	0.0209	0.0000	0.0019	0.0002	0.0004
*S. nodosa*	0.0000	0.0673	0.0062	0.0161	0.0000	0.0014	0.0001	0.0004
*O. fragilis*	0.0000	0.0373	0.0056	0.0102	0.0000	0.0009	0.0001	0.0002
*S. histrica*	0.0000	0.1024	0.0052	0.0223	0.0000	0.0026	0.0001	0.0006
*O. hydroideus*	0.0000	0.0620	0.0052	0.0155	0.0000	0.0016	0.0002	0.0005
*C. aculeata*	0.0000	0.0479	0.0045	0.0118	0.0000	0.0012	0.0001	0.0003
*S. gaarderae*	0.0000	0.0479	0.0032	0.0107	0.0000	0.0012	0.0001	0.0003
*S. corolla*	0.0000	0.0267	0.0023	0.0065	0.0000	0.0005	0.0000	0.0001
*A. acanthifera*	0.0000	0.0224	0.0020	0.0064	0.0000	0.0005	0.0000	0.0001
*Z. hellenica*	0.0000	0.0234	0.0020	0.0064	0.0000	0.0005	0.0000	0.0001
*Papposphaera spp.*	0.0000	0.0197	0.0019	0.0058	0.0000	0.0005	0.0000	0.0001
*Ponthosphaera sp.*	0.0000	0.0287	0.0018	0.0065	0.0000	0.0007	0.0000	0.0002
*S. noroitica*	0.0000	0.0373	0.0018	0.0081	0.0000	0.0009	0.0000	0.0002
*C. oblonga*	0.0000	0.0337	0.0016	0.0073	0.0000	0.0007	0.0000	0.0002
*F. profunda var. elongata XL*	0.0000	0.0173	0.0013	0.0042	0.0000	0.0005	0.0000	0.0001
*S. orbiculus*	0.0000	0.0260	0.0012	0.0057	0.0000	0.0007	0.0000	0.0002
*B. bigelowi*	0.0000	0.0099	0.0009	0.0029	0.0000	0.0002	0.0000	0.0001
*O. reductus*	0.0000	0.0099	0.0009	0.0029	0.0000	0.0002	0.0000	0.0001
*R. parvula*	0.0000	0.0096	0.0009	0.0028	0.0000	0.0002	0.0000	0.0001
*S. ossa*	0.0000	0.0099	0.0009	0.0028	0.0000	0.0002	0.0000	0.0001
*A. acanthos*	0.0000	0.0099	0.0005	0.0022	0.0000	0.0002	0.0000	0.0001
*Neosphaera sp.*	0.0000	0.0089	0.0004	0.0019	0.0000	0.0002	0.0000	0.0001
*S. ossa type 1*	0.0000	0.0087	0.0004	0.0019	0.0000	0.0002	0.0000	0.0001
*S. quadriperforatus*	0.0000	0.0062	0.0003	0.0013	0.0000	0.0002	0.0000	0.0001
*Thoracosphaera spp.*	0.0000	0.1989	0.0369	0.0514	0.0000	0.0035	0.0008	0.0009

### Total coccolith fluxes

The annual mean total coccolith fluxes in the Perdido trap are 62% higher than the flux in the Coatzacoalcos trap, with an annual average flux in Perdido of 3.1 x 10^9^ ± 0.89 x 10^9^ coccoliths per m^-2^d^-1^ and in Coatzacoalcos of 1.9 x 10^9^ ± 1.13 x 10^9^ coccoliths m^-2^d^-1^. Total coccolith fluxes are relatively high through most of the year in Perdido, showing a short-lasting minimum in December, and a trend towards lower fluxes in spring (Fig 4A). Total coccolith fluxes show a higher frequency variability in the Coatzacoalcos trap, showing maximum values of 3.8 x 10^9^ coccoliths per m^-2^d^-1^ in mid-winter, decreasing drastically to a minimum of 0.013 x 10^9^ coccoliths per m^-2^d^-1^ during spring. The normalized fluxes for both traps show a weak seasonality from summer to winter, with higher fluxes during winter and a significant drop during spring, which, in the Coatzacoalcos trap, is linked to a near shutdown of export production (Fig 4B).

### Fluxes of coccolith species assemblages

**Fig 5:**
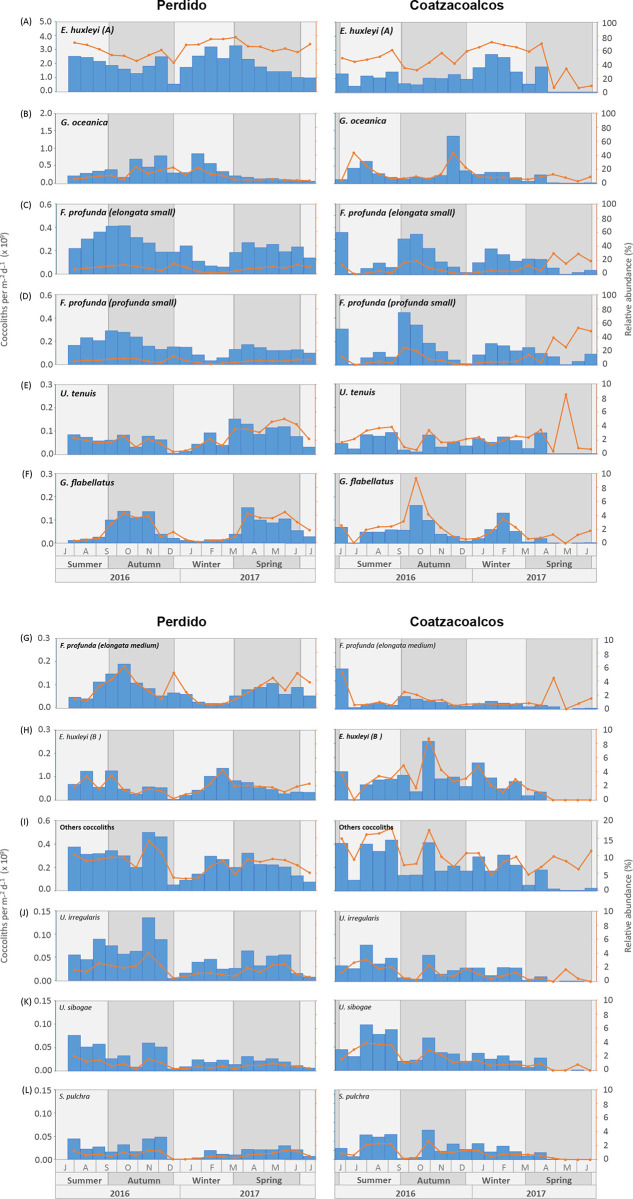
Seasonality of individual species. Coccolith fluxes scale on the left axis (coccoliths per m^-2^ d^-1^ x 10^9^; blue bars) and relative abundances scale on the right axis (%; orange lines) for the Perdido and Coatzacoalcos traps. Light and dark grey bands indicate seasonal periods. Taxa in bold show mean relative abundance >2% throughout the study period, while those <2% were grouped as “Other coccoliths.” Notable contributors to this last group include *U. irregularis, U. sibogae*, and *S. pulchra*. Taxa are sorted in descending order of their relative abundance in the traps. See Table 3 for details.

Species considered as upper photic assemblage (UPZ) taxa comprising *E. huxleyi* type A and B, *G. oceanica*, *G. muellerae*, and *U. tenuis* taxa show the highest fluxes during winter and less so during summer in both locations, and a sharp drop in Coatzacoalcos during spring. This assemblage is mainly composed of *E. huxleyi* type A, with an annual flux of 1.9 x 10^9^ coccoliths per m^-2^d^-1^, followed by *G. oceanica* with 0.3 x 10^9^ coccoliths per m^-2^d^-1^. The fluxes of *E. huxleyi* type B, *G. muellerae*, and *U. tenuis* were less than 0.07 x 10^9^ coccoliths per m^-2^d^-1^ (Fig 5A, B, E, H). The Minimum, maximum, average, and standard deviation for the coccolith fluxes (x 10^9^ coccoliths per m^-2^d^-1^) and their relative abundances (%) for each taxon are presented in Table 3.

The lower photic zone (LPZ) assemblage, include *F. profunda* (var. *profunda* and *elongata*) and *G. flabellatus*, shows higher fluxes during late summer and early autumn and later during spring. The Perdido trap shows the lowest fluxes during winter, *F. profunda* var. *elongata* small was the major contributor to this group, reaching 0.23 x 10^9^ coccoliths per m^-2^d^-1^ on average annually; the contribution of the rest of the species was 0.15 x 10^9^ coccoliths per m^-2^d^-1^. The Coatzacoalcos trap shows relatively high values during the first in late spring of 2016 and low fluxes during the summer; their fluxes increase in early autumn, followed by a significant drop in the autumn-winter transition, and a third small peak in mid-winter, and decreasing towards spring 2017. The species showing the highest was *F. profunda* var. *profunda* small, with an annual average of 0.12 x 10^9^ coccoliths per m^-2^d^-1^ annually (Fig 5C, D, F, G).

The “Other Coccoliths” group includes all species with < 2% relative abundances. Table 3 shows all taxa included in this group for each trap location. Some of the significant contributors to this group were *U. irregularis*, *U. sibogae*, and *S. pulchra*. The Perdido and Coatzacoalcos traps show little seasonal variability in their fluxes. Annual mean values in the Perdido trap for this group reach 0.25 x 10^9^ coccoliths per m^-2^d^-1^. The Coatzacoalcos trap shows high-frequency variability throughout the study period, with an annual average of 0.19 x 10^9^ coccoliths per m^-2^d^-1^, and lowest fluxes toward spring 2017 (Fig 5I, J, K, L).

### Relationship between coccolithophores and environmental parameters

We performed a Spearman correlation analysis to identify the relationship between environmental variability and total coccolith fluxes and the “most common taxa” fluxes. The Perdido trap did not show significant correlations (p < 0.05) between any environmental variable and total coccolith flux. In the Coatzacoalcos trap, positive correlations were observed with Mixed Layer Depth (MLD), Geostrophic Speed (GOS), and negative correlations with Photosynthetically Available Radiation (PAR), Sea Surface Temperature (SST), and Sea Surface Salinity (SSS). In the Perdido trap, the fluxes of *E. huxleyi* type A correlate negatively with Sea Level Anomaly (SLA), while in the Coatzacoalcos trap, it correlates positively with MLD and SLA and negatively with PAR, SST, and SSS. Fluxes of *G. oceanica* correlate positively with MLD and CHL and negatively with PAR and SSS in the Perdido trap, while in Coatzacoalcos, it correlates positively with MLD, GOS, Chl-a, and negatively with PAR. In general, the fluxes of *Florisphaera profunda* (including *profunda* and *elongata* variations) are positively correlated to SLA and SST and negatively to GOS in the Perdido trap, while in Coatzacoalcos, it is positively correlated to MLD and GOS and negatively with PAR. Other coccoliths did not show significant correlations with environmental variables (Table 4).

**Table 4:** Spearman correlation coefficient matrix values of the environmental parameters and the main coccolith fluxes for the Perdido (P) and Coatzacoalcos (C) traps. Each matrix shows the coefficient values in the lower triangle of each section (black color), while the probability values are shown in the upper panel (blue color). Mixed layer depth (MLD), Sea level anomaly (SLA), Geostrophic speed (GOS), Wind speed (WND), PRECIPITATION (PREC), photosynthetically available radiation (PAR), Sea surface temperature (SST), Sea surface salinity (SSS), and Chlorophyll (Chl-a) make up the environmental matrix. Total coccolith flux = Total Flux, *Emiliania huxleyi* type A = *E. hux* (A), *Gephyrocapsa oceanica* = *G. oce*, *Florisphaera profunda* var. *elongata* small = *F. prof* (e s), *F. profunda* var. *profunda* small = *F. prof* (p s), *Umbellosphaera tenuis* = *U. ten*, *Gladiolithus flabellatus* = *G. flab*, *F. profunda* var. *elongata* medium = *F. prof* (e m), Other coccoliths; include the summary of all coccoliths with relative abundances <2%, of which some species are shown. *E. huxleyi* type B = *E. hux* (B), *Umbellosphaera irregularis* = *U. irr*, *Umbilicosphaera sibogae* = *U. sib*, *Syracosphaera pulchra* = *S. pul*, *Calciosolenia brasiliensis* = *C. bra*, *F. profunda* var. *elongata* large = *F. prof* (e l), *Discosphaera tubifera* = *D. tub*, *F. profunda* var. *profunda* medium =* F. prof* (p m).

Trap		MLD	SLA	GOS	WND	PREC	PAR	SST	SSS	Chl-a	Total Flux	*E.* *hux* (A)	*G. oce*	*F. prof* (e s)	*F. prof* (p s)	*U. ten*	*G. flab*	*F. prof* (e m)	Other coccoliths	*E. hux* (B)	*U. irr*	*U. sib*	*S. pul*	*F. prof* (e l)	*F. prof* (p m)
P	MLD		0.32	0.30	0.03	0.19	0.00	0.00	0.00	0.00	0.38	0.39	0.00	0.19	0.42	0.17	0.39	0.18	0.51	0.68	0.59	0.33	0.14	0.45	0.23
P	SLA	−0.24		0.83	0.08	0.04	0.97	0.01	0.43	0.22	0.05	0.00	0.70	0.02	0.07	0.50	0.01	0.00	0.45	0.23	0.11	0.85	0.13	0.27	0.03
P	GOS	0.24	0.05		0.65	0.13	0.28	0.42	0.45	0.96	0.67	0.95	0.44	0.11	0.36	0.21	0.05	0.01	0.92	0.94	0.42	0.76	0.98	0.05	0.04
P	WND	0.48	−0.40	0.11		0.42	0.64	0.00	0.27	0.28	0.57	0.28	0.99	0.08	0.04	0.39	0.52	0.08	0.33	0.57	0.16	0.62	0.33	0.69	0.17
P	PREC	0.31	0.46	0.35	−0.19		0.02	0.98	0.49	0.10	0.11	0.12	0.07	0.98	0.96	0.00	0.90	0.55	0.71	0.36	0.60	0.61	0.59	0.67	0.66
P	PAR	−0.86	0.01	−0.25	−0.11	−0.53		0.05	0.00	0.00	0.34	0.50	0.00	0.74	0.69	0.02	0.52	0.72	0.77	0.82	0.50	0.90	0.33	0.21	0.52
P	SST	−0.76	0.57	−0.19	−0.67	−0.01	0.44		0.02	0.01	0.65	0.28	0.28	0.00	0.00	0.96	0.20	0.02	0.10	0.71	0.06	0.09	0.03	0.79	0.12
P	SSS	−0.70	0.19	−0.18	−0.26	−0.16	0.71	0.53		0.00	0.31	0.33	0.03	0.36	0.71	0.57	0.83	0.51	0.71	0.49	0.97	0.47	0.30	0.31	0.83
P	Chl-a	0.83	−0.29	0.01	0.25	0.37	−0.85	−0.57	−0.80		0.29	0.29	0.00	0.50	0.83	0.10	0.42	0.65	0.40	0.92	0.81	0.31	0.03	0.13	0.83
P	Total Flux	0.21	−0.44	−0.10	0.13	−0.37	−0.23	−0.11	−0.24	0.25		0.00	0.01	0.74	0.77	0.12	0.39	0.09	0.01	0.00	0.06	0.02	0.26	0.75	0.36
P	*E. hux (A)*	0.20	−0.63	0.01	0.25	−0.36	−0.16	−0.26	−0.23	0.25	0.94		0.03	0.23	0.60	0.11	0.09	0.01	0.06	0.00	0.28	0.03	0.46	0.42	0.20
P	*G. oce*	0.62	−0.09	0.18	0.00	0.42	−0.81	−0.26	−0.49	0.68	0.57	0.48		0.83	0.73	0.12	0.24	0.38	0.19	0.35	0.08	0.39	0.77	0.77	0.16
P	*F. prof (e s)*	−0.31	0.53	−0.37	−0.41	0.01	0.08	0.65	0.22	−0.16	−0.08	−0.28	−0.05		0.00	0.87	0.01	0.00	0.07	0.98	0.01	0.09	0.10	0.00	0.04
P	*F. prof (p s)*	−0.19	0.42	−0.22	−0.46	0.01	−0.10	0.62	−0.09	0.05	0.07	−0.12	0.08	0.88		0.75	0.06	0.00	0.10	0.70	0.02	0.11	0.21	0.00	0.04
P	*U. ten*	−0.32	−0.16	−0.29	0.20	−0.62	0.50	−0.01	0.14	−0.38	0.36	0.36	−0.36	0.04	−0.08		0.08	0.92	0.07	0.09	0.23	0.13	0.03	0.76	0.14
P	*G. flab*	−0.20	0.60	−0.44	−0.15	0.03	0.15	0.30	0.05	−0.19	−0.20	−0.39	−0.28	0.58	0.43	0.40		0.00	0.20	0.82	0.03	0.69	0.09	0.00	0.00
P	*F. prof (e m)*	−0.31	0.63	−0.54	−0.40	0.14	0.09	0.50	0.16	−0.11	−0.39	−0.57	−0.21	0.81	0.63	0.02	0.72		0.49	0.25	0.08	0.75	0.38	0.00	0.00
P	Other coccoliths	−0.15	0.18	−0.02	−0.23	−0.09	−0.07	0.37	0.09	−0.20	0.55	0.42	0.31	0.41	0.38	0.42	0.30	0.16		0.02	0.00	0.00	0.00	0.33	0.46
P	*E. hux (B)*	−0.10	−0.28	−0.02	0.14	−0.22	0.05	0.09	0.16	−0.02	0.69	0.72	0.22	0.00	0.09	0.39	−0.05	−0.27	0.50		0.16	0.01	0.25	0.96	0.97
P	*U. irr*	−0.13	0.37	−0.19	−0.32	0.13	−0.16	0.43	0.01	−0.06	0.43	0.25	0.41	0.57	0.50	0.28	0.49	0.40	0.90	0.32		0.00	0.00	0.07	0.15
P	*U. sib*	−0.23	0.04	0.07	−0.12	−0.12	0.03	0.38	0.17	−0.24	0.52	0.48	0.20	0.39	0.37	0.35	0.09	0.08	0.88	0.57	0.74		0.00	0.67	0.72
P	*S. pul*	−0.35	0.35	0.01	−0.23	−0.13	0.23	0.49	0.25	−0.50	0.26	0.17	−0.07	0.38	0.29	0.50	0.39	0.21	0.85	0.27	0.72	0.79		0.73	0.17
P	*F. prof (e l)*	0.18	0.26	−0.45	−0.09	0.10	−0.30	0.06	−0.24	0.35	−0.08	−0.19	0.07	0.67	0.61	0.07	0.61	0.73	0.23	−0.01	0.41	0.10	0.08		0.00
P	*F. prof (p m)*	−0.28	0.47	−0.47	−0.32	0.11	0.15	0.36	−0.05	−0.05	−0.22	−0.30	−0.33	0.47	0.46	0.34	0.81	0.70	0.18	0.01	0.34	0.09	0.32	0.60	
C	MLD		−0.12	0.75	0.81	−0.32	−0.58	−0.75	−0.41	0.32	0.47	0.45	0.47	0.16	0.11	0.32	0.04	0.23	0.28	0.40	0.30	0.28	0.41	0.36	0.60
C	SLA	−0.12		−0.06	−0.25	0.36	−0.36	0.30	−0.43	0.45	0.04	−0.14	0.04	0.44	0.40	−0.07	0.42	0.44	0.04	0.40	0.00	0.05	0.07	0.36	0.29
C	GOS	0.75	−0.06		0.61	−0.21	−0.57	−0.69	−0.48	0.28	0.51	0.48	0.46	0.04	−0.07	0.40	0.05	0.22	0.38	0.52	0.40	0.39	0.49	0.49	0.51
C	WND	0.81	−0.25	0.61		−0.46	−0.32	−0.65	−0.19	0.20	0.28	0.36	0.36	0.08	0.04	0.40	−0.10	0.12	0.24	0.23	0.27	0.24	0.34	0.14	0.45
C	PREC	−0.32	0.36	−0.21	−0.46		−0.28	0.63	−0.18	0.21	−0.05	−0.15	−0.10	0.07	0.14	−0.05	0.29	0.29	0.21	0.38	0.28	0.27	0.20	0.27	0.05
C	PAR	−0.58	−0.36	−0.57	−0.32	−0.28		0.38	0.84	−0.68	−0.54	−0.47	−0.50	−0.35	−0.29	−0.24	−0.41	−0.61	−0.39	−0.71	−0.37	−0.32	−0.41	−0.74	−0.71
C	SST	−0.75	0.30	−0.69	−0.65	0.63	0.38		0.36	−0.03	−0.50	−0.56	−0.36	−0.13	−0.04	−0.27	−0.01	−0.09	−0.16	−0.14	−0.08	0.04	−0.17	−0.27	−0.44
C	SSS	−0.41	−0.43	−0.48	−0.19	−0.18	0.84	0.36		−0.52	−0.44	−0.44	−0.35	−0.35	−0.32	−0.22	−0.45	−0.52	−0.40	−0.61	−0.26	−0.19	−0.40	−0.57	−0.61
C	Chl-a	0.32	0.45	0.28	0.20	0.21	−0.68	−0.03	−0.52		0.43	0.28	0.69	0.20	0.15	0.17	0.29	0.52	0.26	0.52	0.33	0.37	0.31	0.57	0.45
C	Total Flux	0.47	0.04	0.51	0.28	−0.05	−0.54	−0.50	−0.44	0.43		0.94	0.71	0.47	0.29	0.75	0.58	0.55	0.75	0.68	0.61	0.60	0.68	0.66	0.61
C	*E. hux (A)*	0.45	−0.14	0.48	0.36	−0.15	−0.47	−0.56	−0.44	0.28	0.94		0.62	0.44	0.29	0.80	0.56	0.49	0.76	0.61	0.59	0.53	0.66	0.57	0.56
C	*G. oce*	0.47	0.04	0.46	0.36	−0.10	−0.50	−0.36	−0.35	0.69	0.71	0.62		0.00	−0.15	0.63	0.25	0.26	0.58	0.40	0.66	0.65	0.63	0.36	0.30
C	*F. prof (e s)*	0.16	0.44	0.04	0.08	0.07	−0.35	−0.13	−0.35	0.20	0.47	0.44	0.00		0.95	0.24	0.80	0.84	0.33	0.57	0.12	0.19	0.21	0.69	0.74
C	*F. prof (p s)*	0.11	0.40	−0.07	0.04	0.14	−0.29	−0.04	−0.32	0.15	0.29	0.29	−0.15	0.95		0.08	0.77	0.81	0.22	0.48	0.03	0.07	0.10	0.62	0.67
C	*U. ten*	0.32	−0.07	0.40	0.40	−0.05	−0.24	−0.27	−0.22	0.17	0.75	0.80	0.63	0.24	0.08		0.44	0.31	0.91	0.54	0.81	0.79	0.86	0.30	0.34
C	*G. flab*	0.04	0.42	0.05	−0.10	0.29	−0.41	−0.01	−0.45	0.29	0.58	0.56	0.25	0.80	0.77	0.44		0.83	0.62	0.63	0.47	0.48	0.52	0.70	0.65
C	*F. prof (e m)*	0.23	0.44	0.22	0.12	0.29	−0.61	−0.09	−0.52	0.52	0.55	0.49	0.26	0.84	0.81	0.31	0.83		0.51	0.80	0.42	0.44	0.44	0.89	0.82
C	**Other coccoliths**	0.28	0.04	0.38	0.24	0.21	−0.39	−0.16	−0.40	0.26	0.75	0.76	0.58	0.33	0.22	0.91	0.62	0.51		0.70	0.92	0.86	0.94	0.48	0.47
C	*E. hux (B)*	0.40	0.40	0.52	0.23	0.38	−0.71	−0.14	−0.61	0.52	0.68	0.61	0.40	0.57	0.48	0.54	0.63	0.80	0.70		0.65	0.65	0.71	0.81	0.73
C	*U. irr*	0.30	0.00	0.40	0.27	0.28	−0.37	−0.08	−0.26	0.33	0.61	0.59	0.66	0.12	0.03	0.81	0.47	0.42	0.92	0.65		0.91	0.93	0.41	0.38
C	*U. sib*	0.28	0.05	0.39	0.24	0.27	−0.32	0.04	−0.19	0.37	0.60	0.53	0.65	0.19	0.07	0.79	0.48	0.44	0.86	0.65	0.91		0.92	0.42	0.37
C	*S. pul*	0.41	0.07	0.49	0.34	0.20	−0.41	−0.17	−0.40	0.31	0.68	0.66	0.63	0.21	0.10	0.86	0.52	0.44	0.94	0.71	0.93	0.92		0.41	0.45
C	*F. prof (e l)*	0.36	0.36	0.49	0.14	0.27	−0.74	−0.27	−0.57	0.57	0.66	0.57	0.36	0.69	0.62	0.30	0.70	0.89	0.48	0.81	0.41	0.42	0.41		0.84
C	*F. prof (p m)*	0.60	0.29	0.51	0.45	0.05	−0.71	−0.44	−0.61	0.45	0.61	0.56	0.30	0.74	0.67	0.34	0.65	0.82	0.47	0.73	0.38	0.37	0.45	0.84	

**Fig 6:**
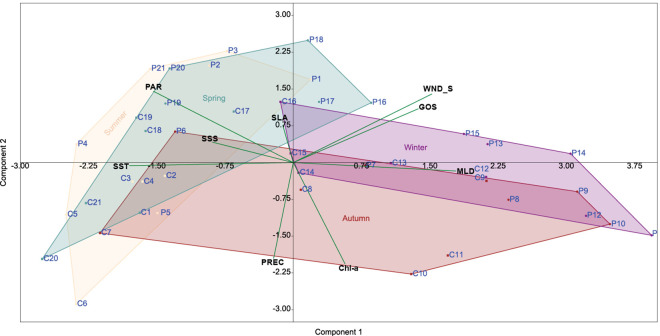
Principal components analysis of environmental parameters. Mixed Layer depth = MLD, sea surface temperature = SST, photosynthetically available radiation = PAR, sea level anomaly = SLA, Wind-speed = WND_S, Precipitation = PREC, Geostrophic speed = GOS, and chlorophyll = Chl-a. Samples are ordinated as a function of the year’s seasons: to the Perdido trap = P (dots) and for the Coatzacoalcos trap = C (squares).

A principal component analysis using environmental data shows that 56% of the total variance within the data set is explained by the first two components, Mixed Layer Depth and Sea Level Anomaly. The first component (PC1) explains 34.4% of the variance and shows positive high loadings mainly with MLD, representative of physical parameters that change with water depth seasonally. The second component (PC2), SLA, explains 21.7% of the variance, is associated with sea level variations produced by cyclonic and anticyclonic eddies, and is positively correlated with high PAR and negatively correlated with precipitation (Table 5). Furthermore, the PCA shows that the samples can be grouped into four groups, which denote the season of the year. Winter and summer show a clear separation, while spring and autumn can be considered transitional seasons. The summer group includes samples from P1 to P5 and P21 for the Perdido trap and samples from C2 to C6 for the Coatzacoalcos; the autumn group includes the samples P6 – P10 and C7 – C11; the winter group included P11- P15 and C12 - C16, finally, the spring group P16 - P20 and C1 and C17 – C21 (Fig 6).

**Table 5:** Principal component factor loadings of environmental parameters.

Component loadings									
Variable	PC 1	PC 2	PC 3	PC 4	PC 5	PC 6	PC 7	PC 8	PC 9
MLD	0.5	0.0	0.3	0.2	0.2	0.4	−0.2	0.4	0.5
SLA	0.0	0.2	0.6	−0.5	0.5	−0.3	0.0	−0.1	0.0
GOS_SPEED	0.4	0.3	0.2	0.0	−0.6	−0.5	0.2	0.1	0.3
WND_SPEED	0.4	0.4	−0.1	−0.1	0.1	0.4	0.6	0.0	−0.3
PRECIPITATION	−0.1	−0.5	0.4	−0.2	−0.3	0.3	0.3	−0.4	0.2
PAR	−0.4	0.4	−0.3	−0.1	0.1	0.1	0.3	−0.2	0.7
SST	−0.5	0.0	0.2	−0.2	−0.2	0.2	0.2	0.8	−0.1
SSS	−0.2	0.1	0.4	0.8	0.1	−0.1	0.2	−0.1	0.0
Chl-a	0.2	−0.5	−0.3	0.0	0.4	−0.4	0.5	0.2	0.2
Variance explained by components	3.7	1.7	1.2	0.9	0.5	0.4	0.3	0.3	0.1
Percent of total variance explained	34.4	21.7	15.0	9.1	7.5	4.8	3.5	3.2	0.9

## Discussion

The two study areas were characterized by a seasonality in the meteorological and oceanographic conditions, highlighting differences between autumn-winter and spring-summer, particularly in the Perdido and to a lesser extent for the Coatzacoalcos trap; these differences affected the production of coccolithophores in the photic zone and consequently the coccolith fluxes recorded in the sediment trap locations. The variability and the magnitude of the coccolith fluxes and oceanographic processes that affected them are discussed in the following sections.

### Seasonal variation of total coccolith fluxes and environmental drivers at the Perdido and Coatzacoalcos traps

The main differences observed in this study are the higher total coccolith fluxes and slightly pronounced seasonality at the Perdido trap compared to Coatzacoalcos. Both locations experienced a significant reduction in export production during spring 2017, particularly at the Coatzacoalcos trap, suggesting a shallower MLD at this site. We believe that this is probably a result of spring and summer time stratification on the water column that can reduce injection of nutrients from deeper layers, limiting essential nutrients for coccolithophore growth, which is more evident in the Coatzacoalcos location. Seasonal stratification in the GoM has been documented using chlorophyll observational data (shipboard and satellite images) and profiling float measurements [37,38,41,43,80], this stratification results from the seasonal increase in temperature during summer and a decrease in the intensity of the wind speed [81] and is characterized by a shallower MLD, with high SST and low Chl-a during spring and summer from the Perdido and Coatzacoalcos areas, as observed in the temporal series. In Fig 2A, B, where the Coatzacoalcos trap shows the lower wind speeds resulting in a shallower mixed layer than the Perdido area, which is further reflected in the total coccolith fluxes. Damien et al. [39] divided the GoM into three regions, where the southwestern or region 1 (including the Coatzacoalcos area) is unproductive and has low chlorophyll during spring because the MLD does not reach the nutrient-rich deep waters, resulting in limited nutrient supply to the surface layer, which is insufficient to support significant phytoplankton growth, while the region 2, central GoM (including the Perdido trap), the Chlorophyll concentration and primary productivity remains relatively stable, and the region 3 (Northern of GoM) shows the highest chlorophyll concentrations for the 3 regions, influenced mainly by the Mississippi River discharges during spring. This limited nutrient supply in the Coatzacoalcos area explains the lower total coccolith fluxes recorded in the trap compared with the Perdido trap, particularly for spring 2017, while the Perdido trap receives nutrients from Region 3 through coastal transport mainly in spring [82,83], as observed with the direction vectors in the mean surface circulation patterns of the GoM, off the coast of Louisiana and Texas U.S.A (Fig 1 A).

**Fig 7:**
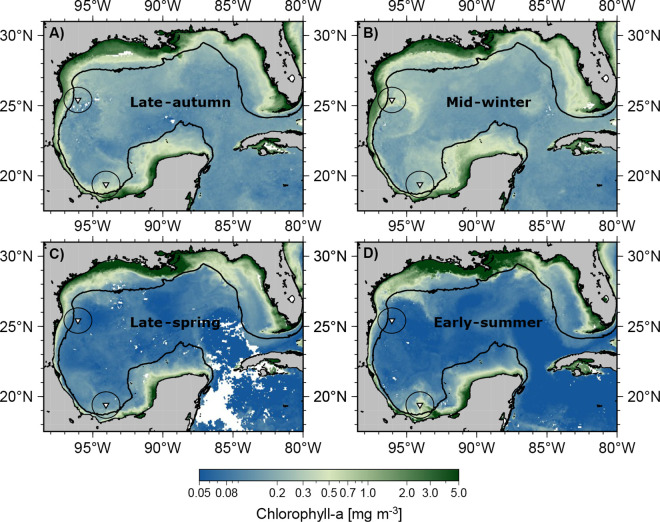
Spatial and temporal variability of sea surface chlorophyll in the GoM. The black circles show the catchment area of each sediment trap. (A) shows the average of the dataset for 11/25/2016 to 12/12/2016 (late autumn), (B) the average from 12/31/2016 to 1/17/2017 (middle winter), (C) shows the average from 5/24/2017 to 6/10/2017 (late spring), and (D) shows the average from 6/29/2017 to 7/16/2017 (early summer). Each panel represents an average of 18 days at 4 x 4 km spatial resolution. https://oceancolor.gsfc.nasa.gov/l3/.

In general, seasonal phytoplankton concentration responds to seasonal changes in the mixed layer deep as a result of the wind stress during winter [37,84], which was reflected in total coccolith fluxes in the sediment traps, showing the highest fluxes from mid-autumn to winter in both areas. However, an evident drop in the Perdido trap in the autumn-winter transition and late February (Fig 4 A, samples 10 and 14) could be the result of the passing of the of Poseidon anticyclonic eddy in the assumed area of influence caught by the sediment trap from early December 2016 as shown with the black contour in the Fig 1B and Fig 2C that shows the SLA variability. Overall, the nutrient distribution in the anticyclonic eddies is influenced by lateral stirring and vertical mixing, with the edge of the eddy having a higher nutrient concentration than the core [85,86]. In the GoM, the nutricline in the Poseidon eddy reached a maximum depth of 250 m during summer, with lower nutrients in the euphotic layer [32,87], limiting phytoplankton growth. During winter, strong winds (Nortes), cold fronts, and proximity to the continental shelf deepen the mixed layer, bringing it closer to the nutricline. This process favors enhanced phytoplankton growth within Eddy Poseidon, as documented by bio-optical properties (in situ fluorescence) measured by a glider [88]. The arrival of Poseidon in the influence area of the Perdido trap with a nutrient-poor core momentarily decreased primary productivity in the trap. Nevertheless, the interaction with the continental shelf and seasonal wind mixing deepened the mixed layer, most likely allowing coccolithophores to grow, which was reflected in the total coccolith fluxes during winter. It has been documented that the Perdido area is periodically affected by the remnant structures of anticyclonic eddies detached from the Loop Current that propagate westwards to the interior of the Gulf [89,90]. When these anticyclonic eddies arrive to the continental shelf (28°- 22°N, 97°- 94°W), the border of these eddies sweeps over the shelf region and advects organic carbon and nutrient-rich waters offshore, forming large filaments that usually extend for 100–200 km offshore in the Perdido area [91]. This is further observed in Fig 1B and Fig 7B, where large chlorophyll filaments reached 200 km offshore in the Perdido area due to the interaction of the Poseidon with the continental shelf.

In the Coatzacoalcos trap, total coccolith fluxes during summer and autumn show a slightly muted variability, most likely driven by the passage of mesoscale cyclonic eddies [30], and offshore transport from the southern continental shelf seems to be important [92]. Cyclonic eddies sweep over the Campeche Bay, advecting coastal nutrients offshore, seasonally impacted by freshwater inputs from the largest river system in the southern Gulf of Mexico, the Grijalva-Usumacinta River complex (Fig 1 A, B) with the highest inputs from summer into autumn rainy season, resulting in chlorophyll plumes and filaments extending tens to hundreds of km offshore [40,92–94] as observed in early summer (Fig 7D). However, these chlorophyll filaments observed in satellite data (Fig 2A) are not reflected in the total coccolith export production at the Coatzacoalcos trap (Fig 4A). This discrepancy likely arises because satellite-derived chlorophyll measurements typically represent only the first optical depth of the column water. Moreover, at the beginning of the study period, the passage of Tropical Storm Danielle (June 19 to June 21, 2016) may have advected material from the continental shelf. In the days following the storm, this could have attenuated light penetration and the primary production, potentially leading to a decrease in the total coccolith flux as observed in Fig 4A (sample 2, Coatzacoalcos). Subsequently, category 1 Hurricane Earl (2–6 August 2026) crossed the area but had a minor impact on the precipitation (Fig 2C).

Annual average coccolith fluxes of the Perdido and Coatzacoalcos traps in the GoM show a similar order of magnitude to the Tropical North Atlantic Ocean, close to the Caribbean Sea. However, they are slightly higher than those recorded in the Cape Blanc and the open equatorial North Atlantic (Table 6). Coccolith fluxes in the Atlantic Ocean show clear spatial gradients in magnitude, species composition, and environmental response, such as oceanographic and atmospheric processes. Generally, changes in primary productivity in the surface waters are well aligned with the reported seasonal variation of the total coccolith fluxes. As recorded in the North Atlantic, the coccolith fluxes are affected mainly by sea surface temperature seasonal changes and mesoscale eddies that affect the primary productivity [13,95,96]. While in the Tropical North Atlantic, stronger stratification conditions influenced by the intertropical convergence zone during summer and autumn result in maximum coccolith fluxes [16]. Species contributions vary with depth and region. Lower photic zone species (e.g., *Florisphaera profunda* and *Gladiolithus flabellatus*) dominate in the western equatorial Atlantic and Caribbean. In contrast, upper photic zone species such as *Emiliania huxleyi* and *Gephyrocapsa oceanica* prevail in NW Africa and the northeast Atlantic [17]. Studies also indicate that nutrient availability, temperature gradients, thermocline depth, wind-forced mixing, and localized inputs (e.g., Amazon River discharge, Saharan dust) influence these fluxes’ magnitude and compositional shifts across the Atlantic [16]. The marine snow and fecal pellets are the primary mechanisms to transport coccoliths to the ocean floor through the water column [5,51,63], while in regions closer to the coast, the contribution of dust from wind and rainfall runoff are the primary mechanisms for sinking coccoliths to the ocean floor [16,97].

**Table 6:** Comparison of maximum coccolith fluxes in different regions. The period of the year in which the maximum flux was recorded, the dominant species, relative abundances, and environmental controls that dominated in the different regions are compared.

Region/ Author	Geographical position (Lat/Long)	Study period	Trap depth (m)	Annual mean coccolith flux (coccoliths m^-2^ y^-1^)	Max flux recorded (coccoliths m^-2^ d^-1^)	Peak flux season	Dominant species throughout the study period	Relative abundance (%)	Environmental drivers
Northeastern Atlantic [95]	48°N, 21° W	April 1989- April 1990	1000	140 x 10^9^	>3.2 x 10^9^	Local Spring bloom period	*Emiliania huxleyi, Gephyrocapsa muellerae and Calcidiscus leptopurus*	86	Cold East Greenland and Labrador Currents. North Atlantic transition zone, influenced by upwelling events induced by mesoscale eddy.
			3700	110 x 10^9^				90	
Northeastern Atlantic [8]	34°N 21°W (NABE-34)	April 1989-April 1990	1000	1.12 x 10^9^	7.5 x 10^9^	Local Spring bloom period	*Emiliania huxleyi*	69	Cold East Greenland, Labrador, and North Atlantic currents. Cyclonic and Anticyclonic eddies. A clear Seasonality in SST and Primary Productivity.
	48°N 21°W (NABE-48)		3700	0.4 x 10^9^	3.2 x 10^9^			72	
North Atlantic [7]	47°N 20°W	18 May 1990−10 June 1990	700	0.16 x 10^9^	3.6 x 10^9^	Local Spring bloom period	*E. huxleyi and Gephyrocapsids, Syracosphaera pulchra (pulse)*		Spring phytoplankton bloom
			1100	0.27 x 10^9^	2.3 x 10^9^				
North Atlantic [9]	31°N 64°W	February 1992-January 1993	3200	1.4 x 10^9^	>2.5 x 10^9^	Late winter-late spring	*E. huxleyi and Florisphaera profunda*	85	Subtropical gyre North Atlantic: Stable surface water stratification through most pof the year, low primary production. Moderate seasonality of of environmental conditions.
Tropical North Atlantic [16]	12°N 49°W (M4)	October 2012 to November 2013	1200	2.41 x 10^9^	4.2 x 10^9^	Mid-autumn	*F. profunda and Gladioluthus flabellatus*	74	Stronger stratification influenced by the Intertropical Convergence Zone during summer. NE trade winds (winter and spring), and the Amazon River discharges (Oct-Nov).
	14°N 37°W (M2)		1200	0.66 x 10^9^	1.5 x 10^9^	Early autumn		69	
Tropical North Atlantic transect [17]	12°N 23°W (M1)	October 2012 to November 2013	1150	0.79 x 10^9^	0.9 x 10^9^	Late winter to spring	*F. profunda and E. huxleyi*	61	The M1 responds to the seasonal changes in stratification and nutrient availability. The CB is influenced by the strong coastal upwelling of Cape Blanc, which enhances nutrient availability and surface productivity during winter and spring.
	21°N 20°W (CB)	October 2012 to February 2014	1214	0.54 x 10^9^	2.15 x 10^9^	Early spring	*E. huxleyi and G. ocenaica*	52	
Souther Gulf of Mexico This study	25° N 96° W (Perdido)	July 2016-July 2017	1050	3.1 x 10^9^	4.29 x 10^9^	Mid-winter	*E. huxleyi, G. ocanica and F. profunda*	85	Loop Current with sporadic spin-off of mesoscale anticyclonic eddies and semi-permanent cyclonic eddy In the Bay of Campeche.
	19° N 94° W (Coatzacoalcos)	June 2016-June 2017	1100	1.9 x 10^9^	3.78 x 10^9^	Mid-winter			

### Oceanographic implications of upper and lower photic zone assemblages

The Upper Photic Zone (UPZ) assemblage was dominant in both traps throughout the study period, reaching 78% for the Perdido trap and 67% for the Coatzacoalcos trap on average. This assemblage includes *E. huxleyi* type A and B, *G. oceanica*, *G. muellerae*, *Umbillicosphaera* spp., *Rabdosphaera* spp., and *Umbellosphaera* spp.

The UPZ dominance throughout the study period reflects the prevalence of coastal processes that inject nutrients into the upper photic zone seasonally and the proximity of the sediment trap deployments to the coast. However, below this zone, they reflect the oligotrophic conditions typical in the deep ocean GoM. Under these conditions, the phytoplankton groups are limited by nutrient availability in the upper euphotic zone and light intensity in the lower euphotic zone [98]. During summer and early autumn 2016 and late spring 2017, surface warming and reduced wind activity generated a shallow mixing layer as observed with SST and MLD in (Fig 2A, B), limiting the upwelling of nutrients from the thermocline and showing a high stratification [37,39,41].

**Fig 8:**
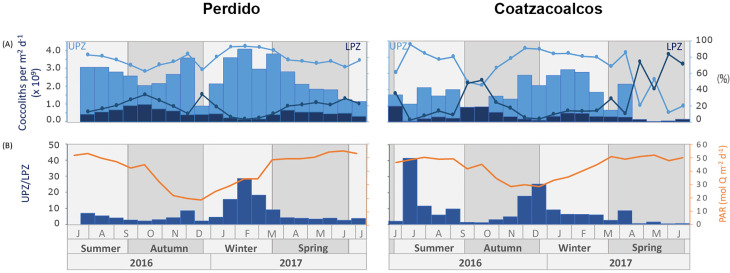
Seasonality of upper and lower photic assemblages for the Perdido and Coatzacoalcos traps. (A) Relative abundances of the Upper Photic Zone (UPZ) (blue lines with dots) and relative abundances for the Lower Photic Zone (LPZ) (dark blue lines with dots), respectively. Export fluxes of UPZ assemblages (blue bars), and LPZ assemblages (dark blue bars). (B) The UPZ/LPZ ratio (dark blue bars) and photosynthetically available radiation (PAR) (mol Q m^-2^ d^-1^) (orange solid line).

Mean fluxes were higher in the Perdido trap (2.47 x 10^9^ ± 0.89 x 10^9^ coccoliths per m^-2^d^-1^) than in the Coatzacoalcos trap (1.45 x 10^9^ ± 0.97 x 10^9^ coccoliths per m^-2^d^-1^) (Fig 8A). This difference in the magnitude of mean fluxes of the UPZ reflects the lower nutrient injection in the Coatzacoalcos than in the Perdido area, as documented by Damien et al. [39], with variability of chlorophyll concentration in the GoM.

Seasonal variability of the UPZ fluxes responds to nutrient injection associated with the seasonal influence of coastal processes, such as filaments and plumes produced by the river discharges that inject nutrients into the deep ocean of the GoM, advise this stratification, too [40,92,94,99], and the interaction of the mesoscale cyclonic and anticyclonic eddies with the coast [25,30,89], as mentioned in the previous section.

Furthermore, during the winter season, the major activity in the Nortes winds deepens the mixed layer, reaching the nutricline, promoting primary productivity in both areas [39,44,92] and the coccolith export production. Nutrient availability in surface waters plays a critical role in structuring coccolithophore communities as observed in the variation of UPZ/LPZ ratio (Fig 8B). This nutrient injection is consistent with studies demonstrating that this species thrives in environments influenced by nutrient pulses from coastal upwelling, riverine discharge, and mesoscale eddies [100–102]. We used the UPZ/LPZ ratio as a proxy of nutricline depth and believe that the nutrient injection could be in short temporal pulses, as observed in the UPZ/LPZ ratio (Fig 8B) that are related to more productive UPZ regions, where the nutricline is shallower, while low values are associated with deeper nutricline [3,16,17,66,68].

The Lower Photic Zone (LPZ) assemblages are composed of *G. flabellatus*, *F. profunda* var. *profunda* (small and medium), and var. *elongata* (small, medium, and large) exhibited a more stable presence with lower fluxes: mean of 0.54 x 10^9^ ± 0.24 x 10^9^ coccoliths per m^-2^ d^-1^ in the Perdido trap and 0.33 x 10^9^ ± 0.28 x 10^9^ coccoliths per m^-2^ d^-1^ in Coatzacoalcos (Fig 6 A). In the Perdido trap, LPZ averaged 19% throughout the study period, while in the Coatzacoalcos trap, it accounted for 21%. The relatively higher LPZ abundances in Coatzacoalcos, particularly in the early autumn of 2016 and late spring of 2017, coincided with increased precipitation (Fig 2C and Fig 8A) and riverine discharge [40,103], suggesting a link between deep chlorophyll maxima and nutrient availability at depth. The peak contribution of LPZ species in spring 2017 supports the hypothesis of a transient deepening of the nutricline, as suggested by Guerreiro et al. [16] in their study of sediment traps in the Amazon-influenced Atlantic.

Studies in the Equatorial Atlantic [16,17,65] and the Bay of Bengal [68] indicate that increased LPZ fluxes typically signal oligotrophic conditions with a deep nutricline, where light availability rather than nutrients becomes the primary limiting factor. This mainly explains why the Coatzacoalcos trap shows a higher relative abundance of the LPZ during spring 2017, as it is more strongly influenced by coastal stratification and seasonal runoff. Several ecological studies have documented that the UPZ taxa were mostly influenced by temperature and the availability of nitrate and phosphate. In contrast, the LPZ assemblages are most likely limited by light availability [2,3,64]. Sediment trap studies have reported UPZ assemblages with high abundances and fluxes in regions influenced by coastal upwelling systems, such as the Cape Blanc region off NW Africa [14,15,17], the Bay of Bengal by [68], and the Southern California Current [97]. This is reflected in the shallowing of the nutricline and low fluxes observed in the LPZ assemblages [66,102]. The results we have found in the traps deployed within the GoM during the winter season highlight the importance of multifactorial and seasonal nutrient injections and the response of coccolithophore assemblages.

### Coccolithophore assemblages

The dominance of *Emiliania huxleyi* and the high abundance of *Gephyrocapsa oceanica* throughout the study period in both areas reflect their opportunistic adaptation in dynamic coastal environments [2,65,100,104–106].

*E. huxleyi* is the cosmopolitan coccolithophore, one of the most euryhaline and eurythermal species [63], occurring at a relative abundance of 60–80%, mainly in the southern high-latitude regions [107,108]. However, their abundance and fluxes are low in larger parts of the open ocean, such as the subtropical gyres [2,3,17]. *G. oceanica*, which may also be dominant in warm marginal seas as a typical Atlantic coastal upwelling [109,110] and also found in upwelling areas like the Northeastern Arabian Sea, is associated with nutrient injection during monsoon periods [111]. In the GoM, the highest relative abundances and fluxes of *E. huxleyi* and *G. oceanica* are associated with the variability of mixed layer depth, which deepens in late autumn and during winter, following the seasonal pattern of the Nortes winds [44]. The species *Florisphaera profunda* (including the variation *profunda* and *elongata*; Fig 5C, D, G) and *G. flabellatus* (Fig 5F) showed a seasonal pattern with high relative abundances and fluxes in the summer-autumn transition and spring during the highest stratification (Fig 2A; SST). Several studies [2,3] report these taxa to be most abundant in the LPZ of the subtropical gyres, where the stratification is derby, allowing the necessary stable condition for light to penetrate deeper into the photic zone, this reflects the adaptation to high stratification and lower light intensities (~1% light) of this species [8,17,64,112]. *Umbellosphaera tenuis*, *U. irregularis*, and *Umbilicosphaera sibogae*, tropical species (Fig 5E, J) also increased during this same period and appear to be related to slightly higher PAR levels (Fig 6 B) and stratification by the SST (Fig 2A), which is consistent with other studies about of coccolithophores ecology [2,16,64,65,112]. In general, *Syracosphaera* *pulchra* showed the highest fluxes during summer and autumn with some drops (Fig 5L), suggesting the greatest preference for warm, oligotrophic conditions [106,113].

### Ecological dynamics of coccolithophores in the GoM

**Fig 9:**
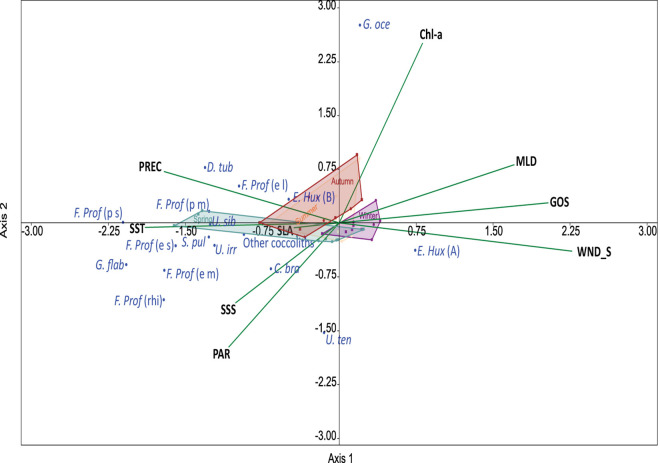
Ordination diagram of the canonical correspondence analysis (CCA) for the Perdido and Coatzacoalcos traps. Performed with the environmental variables (MLD, SLA, GOS, WND_S, PREC, PAR, SST, SSS, Chl-a) that were averaged over the 18 days for each cup opening (July 2016 to June 2017) and the principal coccolith fluxes: *Emiliania huxleyi* type A = *E. hux* (A), *Gephyrocapsa oceanica* = *G. oce, Florisphaera profunda* var. *elongata* small = *F. prof* (e s), *F. profunda profunda* small = *F. prof* (p s), *Umbellosphaera tenuis* = *U. ten, Gladiolithus flabellatus *= *G. flab, E. huxleyi* type B = *E. hux* (B), *F. profunda elongata* medium = *F. prof* (e m), and Others coccoliths group *Umbellosphaera irregularis* = *U. irr*, *Umbilicosphaera sibogae* = *U. sib*, *Syracosphaera pulchra* = *S. pul, Calciosolenia brasiliensis* = *C. Bra, F. profunda* var. *elongata* large = *F. prof* (e l), *Discosphaera tubifera* = *D. tub*, *F. profunda profunda* medium = *F. prof* (p m), *F. profunda* var. rhinocera = *F. prof (rhi)*. The colors of polygons indicate the seasons of the year and are formed by each of the samples (points).

Throughout this study, the coccolithophore assemblages recorded in sediment traps in the Southwestern Gulf of Mexico reflect the primary productivity pulses and the related environmental processes. Canonical correspondence analysis (CCA) revealed that key environmental parameters such as Mixed Layer Depth (MLD), Sea Level Anomaly (SLA), and Geostrophic speed (GOS) explained 92% of the variance in coccolith fluxes, underscoring the strong coupling between physical and biological response in both the Perdido and Coatzacoalcos areas (Fig 9). The eigenvalues and species-environment correlation coefficients provide quantitative measures of the importance of each axis and the strength of the relationship between species and environmental variables [79], as shown in Table 7.

**Table 7:** Canonical correspondence analysis. Environmental variables, eigenvalues, variance (%), and permutation values for the data set of the Perdido and Coatzacoalcos traps.

Variable	Eigenvalue	%	p
MLD	0.0434	44.54	0.004
SLA	0.0345	35.45	0.001
GOS	0.0119	12.2	0.008
WND_S	0.0032	3.287	0.164
PREC	0.0020	2.011	0.066
PAR	0.0017	1.786	0.001
SST	0.0005	0.5632	0.091
SSS	0.0002	0.1668	0.345
Chl-a	6.33 x 10^-11^	6.49 x 10^-08^	0.992

In winter, intensified wind forcing deepens the MLD [43], enhancing nutrient entrainment into the upper photic zone [39,93]; this is primarily associated with cold wind fronts (Nortes) during autumn-winter, contributing to strong geostrophic currents [44], and increasing the chlorophyll concentration during this period, as shown in the spatial distribution of Chl-a (Fig 7A, B), variation of environmental conditions (Fig 2A, B), and the CCA with positive values in this environmental parameter (Fig 9). These environmental conditions favor r-strategic coccolithophores, particularly *E. huxleyi* and *G. oceanica*, which thrive in nutrient enrichment, mixed waters commonly found over continental margins [63,114]. Their dominance during this period aligns with their ecological traits as fast-growing, opportunistic taxa [4]. In the GoM, this was reflected in a decrease in richness and diversity indices from the coccolithophore species (Fig 3A).

On the other hand, the spring and summer seasons are characterized by a strong stratification with elevated sea surface temperatures, a shallow MLD as observed in the variation of environmental conditions (Fig 2A, B), and the spatial distribution of Chl-a (Fig 7C, D), and nutrient depletion (UPZ/LPZ ratio, Fig 8B). Under these conditions, k-strategic coccolithophore species inhabiting the UPZ flourish (*U. tenuis, D. tubifera*, and *Rhabdosphaera* spp.) as documented in several plankton studies in the North Atlantic Ocean [2,112]. This agrees with the results found in our study, where the species *F. profunda, U. tenuis, U. irregularis, U. sibogae, C brasiliensis, E. huxleyi* type B, *F. profunda, G. flabellatus*, and the group “Other coccoliths” are grouped to the spring-summer season, mainly associated with SST, SSS, and PAR (Fig 9).

Our results show that the LPZ assemblages, particularly *F. profunda* and *G. flabellatus*, showed a strong correlation with photosynthetically available radiation (PAR, p = 0.001) and a marginal association with Precipitation (p = 0.06) (Table 4). Both species have been recorded as restricted to light in the lower euphotic zone in the tropical and subtropical oceans [2,3,65]. The dominance of LPZ assembles during periods of strong stratification (e.g., summer and autumn) indicates their ecological importance in maintaining productivity in oligotrophic (nutrient-poor) environments like the western North Atlantic Ocean [16]. However, low LPZ fluxes and the dominance of the UPZ in both sediment trap deployments reflect mainly the nutrient injection from the coast by lateral advection and sweep of nutrient and organic carbon from the shelf to the offshore, as discussed previously. Although the Coatzacoalcos area proved to be the most oligotrophic in the Perdido area, this can be observed in the higher relative abundance of the Coatzacoalcos trap, which increased particularly in spring 2017 (Fig 8A). During stratification periods, the limited nutrients available in the euphotic zone are recycled more rapidly, giving smaller cells a competitive advantage over larger ones [115]. Consequently, Pico and nannoplankton, such as coccolithophores (k-Selected: e.g., *U. irregularis, D. tubifera, R. clavigera*), dominate these systems and form the foundation of the food chain, as low nutrient levels do not restrict coccolithophores growth [116,117], as recorded recently in the subtropical gyre in the North Atlantic [2,3]. Observations from previous studies have shown that when resources are extremely limited (low nutrient conditions), coccolithophores can absorb organic compounds by mixotrophy [2,118,119] and osmotrophy [120,121], a strategy that may play an important role during strong stratification (spring-summer) in the deep ocean of the GoM.

## Conclusion

This study examines the seasonal variability of coccolith fluxes in the Perdido (western GoM) and the Coatzacoalcos area (southwestern GoM) and identifies the environmental factors most likely influencing these fluxes.

The Perdido trap recorded less species richness, with 47 species of coccoliths, compared to the Coatzacoalcos trap with 56 species throughout the study period. However, the Perdido trap showed higher total coccolith fluxes with an annual average of 3.1 x 10^9^ ± 0.89 x 10^9^ coccoliths per m^-2^d^-1^, than the Coatzacoalcos average of 1.9 x 10^9^ ± 1.13 x 10^6^ coccoliths m^-2^d^-1^.

Three species dominated the composition of the coccolith sinking assemblages in both areas: *E. huxleyi, G. oceanica*, and *F. profunda*, reaching 88% in the Perdido and 84% in the Coatzacoalcos area.

Total coccolith fluxes showed a seasonal response in both trap locations with lower fluxes during spring and summer, associated with well-stratified water column conditions, and higher fluxes during late autumn and winter, associated with the deepening mixed layer in response to cooling and to the strong Nortes winds. Both trap locations showed a reduction during the spring of 2017, associated with seasonal stratification, more evident in the Coatzacoalcos area due to higher stratification in the Southwestern region that limited the nutrient supply to the surface layer, which is insufficient to support significant phytoplankton growth, as has been described by Damien et al. [39].

The mesoscale eddies further play an important role in the modulation of fluxes in the GoM: In the Perdido area, the passage of the Poseidon anticyclonic eddy is associated with decreased coccolith fluxes temporarily (autumn-winter transition) when the core arrives at the influence area of the Perdido trap.

The ecological index shows higher richness and diversity in the Coatzacoalcos region than in the Perdido region; both regions presented low evenness, suggesting the dominance of a few species.

The upper photic zone (UPZ) assemblage dominated throughout the study period in both regions and most likely reflects the sporadic pulses of nutrients from the coastal shelf to the deep ocean. We used the UPZ/LPZ ratio as a proxy for the variability of the nutricline. The highest ratios were related to a deeper mixed layer, and most likely mixing with nutrient-rich surface waters, resulting in higher coccolith fluxes during winter in both regions; these environmental conditions favor r-strategic coccolithophores, particularly *E. huxleyi* and *G. oceanica*, that are commonly found over continental margins.

Moderate fluxes of total coccolith fluxes during summer-autumn reflect upper ocean stratification and, most probably, low nutrient availability in the upper photic zone. Under these conditions, k-strategic coccolithophore species inhabiting the LPZ flourish (*F. profunda, G. flabellatus, U. tenuis, D. tubifera,* and *Rhabdosphaera* spp., and other coccoliths).

These results suggest that coccolithophore export production in the Perdido and Coatzacoalcos traps is strongly influenced by cooling and deepening of the MLD during autumn and winter, as well as advection processes between the continental shelf and the offshore region, and multi-factorial processes such as loop current mesoscale eddies.

## Supporting information

S1 File Inclusivity-in-global-research-questionnaire. (DOCX)

S1 Table Time series data of atmospheric and oceanographic parameters. Dataset downloaded from satellite imagery and models at the sea surface for the catchment area of the sediment trap (1° radius around each sediment trap).
(PDF)

S2 Table An Excel file containing all data is supplied with this article. It shows the dates of each deployment, sample ID, satellite data and models, ecological indices, total coccolith flux (coccoliths per m-2d-1), normalized flux, and relative abundance and flux of the most common species.
(XLSX)
