# Supplementary material for: Seasonal variability of coccolith fluxes in sediment traps of the Perdido and Coatzacoalcos regions in the Southern Gulf of Mexico
Source: PLoS One. 2025 Nov 5;20(11):e0326673. doi: 10.1371/journal.pone.0326673 (PMC12588537; doi:10.1371/journal.pone.0326673)
Supplement: S1 Table — Dataset downloaded from satellite imagery and models at the sea surface for the catchment area of the sediment trap (1° radius around each sediment trap). (PDF) [file pone.0326673.s002.pdf]

S1 Table 1. Time series data of atmospheric and oceanographic parameters. Dataset downloaded from satellite imagery and models at the sea surface for the catchment area of the sediment trap (1° radius around each sediment trap).

Variable	Product name	Spatial resolution	Temporal resolution	Main URL	Reference
**SST**	Optimally Interpolated Sea Surface Temperature version 2.1	0.25°x0.25°	Daily	https://www.ncei.noaa.gov/thredds/dodsC/OisstBase/NetCDF/V2.1/AVHRR/	Huang *et al.* , (2021)
**PAR**	Photosynthetically Active Radiation (PAR) estimated from the EPICS-DISCOVR dataset	18x18 km	Daily	https://epic.gsfc.nasa.gov/	Frounin *et al.,* (2018)
**Chl-a**	MODIS-Aqua surface chlorophyll from the OCI algorithm	4x4 km	Daily	https://oceancolor.gsfc.nasa.gov/	Hu *et al.,* (2012)
**MLD**	Mixed Layer Depth from density estimated from the HYCOM model analysis outputs for the Gulf of Mexico (expt 32.5)	0.04°x0.04°	Daily (at 00Z hrs)	https://www.hycom.org/data/goml0pt04/expt-32pt5	Metzeger et al., (2014)
**Wind speed**	Ocean surface winds from the<br><br>Cross- Calibrated Multiplatform variational analysis (CCMP version 3.0)	0.25°x0.25°	6 hours	https://data.remss.com/ccmp/	Mears *et al.,* (2022), *Atlas et al.,* (2011)
**PREC**	Precipitation from the Integrated Multi-Satellite Retrievals for GPM (IMERG) algorithm	0.25°x0.25°	Daily	https://gpm1.gesdisc.eosdis.nasa.gov/	Huffman *et al.,* (2020)
**SLA**	Sea Level Anomalies Global Ocean Gridded L 4 Sea Surface Heights and Derived Variables Reprocessed 1993 Ongoing	0.25°x0.25°	Daily	https://data.marine.copernicus.eu/	CMEMS https://doi.org/10.48670/moi-00148

BIBLIOGRAPHY

1.

Atlas, R., Hoffman, R.N., Ardizzone, J., Leidner, S.M., Jusem, J.C., Smith, D.K. *et al.* (2011). A Cross-calibrated, Multiplatform Ocean Surface Wind Velocity Product for Meteorological and Oceanographic Applications. *Bulletin of the American Meteorological Society*, 92, 157-174.

2.

Frouin, R., Tan, J., Ramon, D., Franz, B. & Murakami, H. (2018). *Estimating photosynthetically available radiation at the ocean surface from EPIC/DSCOVR data*. SPIE.

3.

Hu, C., Lee, Z. & Franz, B. (2012). Chlorophyll algorithms for oligotrophic oceans: A novel approach based on three-band reflectance difference. *Journal of Geophysical Research: Oceans*, 117.

4.

Huang, B., Liu, C., Banzon, V., Freeman, E., Graham, G., Hankins, B. *et al.* (2021). Improvements of the Daily Optimum Interpolation Sea Surface Temperature (DOISST) Version 2.1. *Journal of Climate*, 34, 2923-2939.

5.

Huffman, G.J., Bolvin, D.T., Braithwaite, D., Hsu, K.-L., Joyce, R.J., Kidd, C. *et al.* (2020). Integrated Multi-satellite Retrievals for the Global Precipitation Measurement (GPM) Mission (IMERG). In: *Satellite Precipitation Measurement: Volume 1* (eds. Levizzani, V, Kidd, C, Kirschbaum, DB, Kummerow, CD, Nakamura, K & Turk, FJ). Springer International Publishing Cham, pp. 343-353.

6.

Mears, C., Lee, T., Ricciardulli, L., Wang, X. & Wentz, F. (2022). Improving the Accuracy of the Cross-Calibrated Multi-Platform (CCMP) Ocean Vector Winds. *Remote Sensing*, 14, 4230.

7.

Metzger, E.J., Ole Martin, S., Prasad, G.T., Harley, E.H., James, A.C., Alan, J.W. *et al.* (2014). US Navy Operational Global Ocean and Arctic Ice Prediction Systems. *Oceanography*, 27.

This study has been conducted using E.U. Copernicus Service Information; CMEMS https://doi.org/10.48670/moi-00148 (23/11/2023: accessed date).
